# Combined Genetic and Telemetry Data Reveal High Rates of Gene Flow, Migration, and Long-Distance Dispersal Potential in Arctic Ringed Seals (*Pusa hispida*)

**DOI:** 10.1371/journal.pone.0077125

**Published:** 2013-10-10

**Authors:** Micaela E. Martinez-Bakker, Stephanie K. Sell, Bradley J. Swanson, Brendan P. Kelly, David A. Tallmon

**Affiliations:** 1 Department of Ecology and Evolutionary Biology, University of Michigan, Ann Arbor, Michigan, United States of America; 2 Biology and Marine Biology Program, University of Alaska Southeast, Juneau, Alaska, United States of America; 3 Department of Biology, Central Michigan University, Mount Pleasant, Michigan, United States of America; 4 Arctic Sciences Section, National Science Foundation, Arlington, Virginia, United States of America; University of Otago, New Zealand

## Abstract

Ringed seals (*Pusa hispida*) are broadly distributed in seasonally ice covered seas, and their survival and reproductive success is intricately linked to sea ice and snow. Climatic warming is diminishing Arctic snow and sea ice and threatens to endanger ringed seals in the foreseeable future. We investigated the population structure and connectedness within and among three subspecies: Arctic (*P. hispida hispida*), Baltic (*P. hispida botnica*), and Lake Saimaa (*P. hispida saimensis*) ringed seals to assess their capacity to respond to rapid environmental changes. We consider (a) the geographical scale of migration, (b) use of sea ice, and (c) the amount of gene flow between subspecies. Seasonal movements and use of sea ice were determined for 27 seals tracked via satellite telemetry. Additionally, population genetic analyses were conducted using 354 seals representative of each subspecies and 11 breeding sites. Genetic analyses included sequences from two mitochondrial regions and genotypes of 9 microsatellite loci. We found that ringed seals disperse on a pan-Arctic scale and both males and females may migrate long distances during the summer months when sea ice extent is minimal. Gene flow among Arctic breeding sites and between the Arctic and the Baltic Sea subspecies was high; these two subspecies are interconnected as are breeding sites within the Arctic subspecies.

## Introduction

Warming climate is an imminent threat to the persistence of Arctic fauna [1], [2]. The unprecedented melting rate of Arctic sea ice has resulted in elevated mortality of ice-adapted marine mammals such as the polar bear and ringed seal [3]–[6]. The potential of these species to adapt to their changing environment will depend largely upon the spatial structure of their populations and the amount of gene flow [7]. If a species consists of many geographically isolated populations with low levels of gene flow, it will have low realized genetic variation, which may reduce the efficiency of natural selection and lead to the fixation of non-adaptive traits [7]–[9]. Assessing a species’ capacity to respond to global climate change requires knowledge of its population structure and spatial partitioning of genetic variation. Here we investigate the population structure, migration, and use of sea ice by ringed seals (*Pusa hispida*). In particular, we focused on three of the five subspecies: the Arctic subspecies (*P. h. hispida*), the Baltic Sea ringed seal (*P. h. botnica*), and the subspecies landlocked in Lake Saimaa, Finland (*P. h. saimensis*) which are listed as threatened (Arctic and Baltic subspecies) or endangered (Lake Saimaa subspecies) under the U.S. Endangered Species Act [10], [11]. We quantified gene flow between breeding sites (i.e. tentative populations) and dispersal potential, essential parameters in developing effective conservation strategies.

Ringed seals are the most abundant marine mammal in the Arctic with a nearly continuous distribution in the Arctic Ocean [12]. They are important in the diet of Arctic carnivores, including imperiled polar bears [13], Arctic foxes [14], and indigenous Arctic people [15], [16]. The overall population size is unknown but generally thought to be several million. The species has historically been viewed as robust and only marginally impacted by stressors such as predation, human harvesting, ecotoxins, or disease [6]. Ringed seal abundance can be directly attributed to adaptation to the great expanse of Arctic sea ice [17], but their dependence on sea ice may now be maladaptive in the face of global climate change.

The reproductive success of ringed seals is contingent upon the accumulation of snow atop Arctic sea ice, but snow cover is diminishing on Arctic sea ice and is forecast to be insufficient for rearing young over most of the range by the end of the century [18]. Both males and females haul out onto the sea ice in subnivean lairs excavated as early as February, and probably much earlier when snow is sufficient. Whelping and nursing take place within lairs from March-June. Subnivean lairs are adequate to shelter ringed seals from predators and temperatures as low as −61°C when snow depth exceeds 45 cm, a condition only found in locations where large drifts have formed [6]. Over much of the Arctic Ocean, annual freeze-up is increasingly delayed, and a greater fraction of the season’s snow falls in to the open water. As a result, less snow accumulates on the ice, further diminishing pupping habitat. The Saimaa ringed seal was listed as endangered in 1993 due to low census size and habitat deterioration [19]. The remaining subspecies were listed as threatened in 2012 due to diminished snow cover, hypothermia-induced-mortality from premature melting of lairs, and climate model projections of accelerated sea ice habitat loss [6], [10], [20]. The once predictable environment to which they have adapted is now subject to increasing inter-annual variation in ice and snow cover, jeopardizing reproductive success and persistence of the species [1], [6]. We investigated genetic variation and spatial structure of ringed seal populations to evaluate their susceptibility to population declines and local extinction driven by diminishing snow and ice cover.

Previous molecular studies, using many of the same genetic markers used in this study, found minimal genetic differentiation among tentative ringed seal populations. Palo et al. [21] investigated the genetic differentiation among Arctic and Baltic ringed seals sampled from Svalbard and Spitsbergen, the Gulf of Bothnia, and the Gulf of Finland [21]. Baltic populations were indistinguishable from each other (F_ST_ = 0.000 based on eight microsatellite loci) and the differentiation between the Baltic and Arctic subspecies was weak (F_ST_ = 0.017). Likelihood-based inference suggested 1–2 migrants from the Arctic into the Baltic per year on average (i.e. nine effective immigrants per generation). Estimated levels of immigration into the Baltic has been sufficient to prevent high levels of genetic differentiation between the subspecies, but is insufficient for countering population decline [21]. In 2003, Palo et al. estimated the level of genetic differentiation between Saimaa and Arctic pooled with Baltic ringed seals. They found a 69% reduction in microsatellite diversity in the Saimaa ringed seal compared to the other subspecies and inferred that the loss of diversity was due to a low effective population size (N_e_ ∼ 350). They also found little genetic differentiation among breeding sites within Lake Saimaa (F_ST_ = 0.02) [22], similar to the findings in the Baltic.

Davis et al. [23] estimated the amount of genetic differentiation among Arctic ringed seals sampled from eight geographical locations: the Bering Sea, the Beaufort Sea, Hudson Bay, Frobisher Bay, Grise Fjord, the west coast of Greenland, Svalbard, and the White Sea. With the exception of the White Sea, pairwise measures of differentiation among sample sites suggested little regional differentiation within the Arctic subspecies (F_ST_ range from 0.0000–0.0041). Moderate differentiation was found between the White Sea and all other sample sites (F_ST_ range from 0.0180–0.0306). Bayesian inference of population structure, however, suggested individuals from all eight locations belong to a single panmictic population.

Palo et al. and Davis et al. collected samples throughout the year, with many of their samples collected outside of the breeding season when many ringed seals travel far from their breeding sites [24]. Swanson et al. [25] highlighted the importance of collecting genetic samples in breeding sites, and they detailed a method for doing so. We restricted our sampling to seals in their breeding sites. We used two mitochondrial regions and nine microsatellite loci to measure the amount of gene flow among nine breeding sites of Arctic ringed seals as well as the Saimaa and Baltic subspecies. Our sample sites are distinct from those studied by Palo [21], [22] and Davis et al. [23], yet span a similar geographic range. In addition to our molecular analysis, we used satellite-telemetry to analyze the seasonal movements of ringed seals. We also compared the seals’ use of sea ice at two different Arctic breeding locations to test for localized differences in haulout patterns between breeding populations.

Identifying regional variation in haulout behavior is important for understanding how ringed seals utilize the ice environment and Arctic food resources. Ringed seal haulout and diving activity provides insights into interactions with other trophic levels, with diel diving behavior linked to seasonal activity budget and the vertical distribution of prey items [26]. The abundance and distribution of ringed seals is attributed to their highly variable feeding habits and diverse prey items, the foremost of which are: arctic, polar, and saffron cod, Decapods, Euphausiids, and large Amphipods [27]–[29]. The haulout season is a time of intense fasting and declining body condition [29]. Ringed seals digest their food quickly; thus, the stomachs of dry hauled-out seals are assumed empty [27]. Hauling out also makes ringed seals susceptible to polar bear predation and visible for aerial surveys.

By combining direct observations of behavior (satellite-telemetry) with indirect measurements of gene flow (population genetic analysis), we provide novel insight into ringed seal population ecology that can inform management decisions. Specifically, we asked: (1) what is the geographical scale at which seals migrate? (2) Is there variation in the behavior of seals from different breeding sites? (3) How much gene flow is there between ringed seal subspecies? And (4) how much gene flow is there between breeding populations of Arctic ringed seals?

## Results

### Migration and Behavioral Differences among Breeding Sites

We tracked 27 ringed seals (n_Male_ = 14, n_Female_ = 13) from four breeding sites in Western Alaska and Canada to directly quantify movements. Satellite-linked tags attached to the seals’ rear flippers reported their locations via the Argos satellite system for periods of a few days to 13 months. After censuring unreliable locations, we had from 4 to 113 locations per seal (Table 1). Of the 27 seals tagged, 9 adults travelled over 400 km from their breeding site, and 4 of them moved over 1000 km. All long distance movements occurred between April and November (Figures S1–S16).

**Table 1:** Satellite telemetry results for 27 seals.

Seal	Sex/Age	Capture	Last location	No. obs.	Ice bound dist. (mean, max)	Open water dist.(mean, max)	pval
AF06	F/7	5/6/06	2/12/07	113	(4.4, 21.8)	(14.8, 76.1)	0.000
AS05	F/6	5/6/05	3/15/06	52	(11.2, 24.1)	(21.4, 173.1)	0.239
BF06	F/7	5/14/06	1/5/07	63	(4.9, 15.0)	(16.1, 145.8)	0.297
IO05	F/4	5/9/05	7/13/05	32	(4.3, 11.5)	(4.8, 14.3)	0.830
LY05	F/7	5/4/05	1/22/06	43	(8.0, 45.8)	(13.9, 155.0)	0.669
ME08	F/5	5/25/08	8/3/08	36	(1.4, 2.3)	(37.3, 107.7)	0.016
Pak0605	F/6	3/23/06	6/25/06	10	(300.5, 1667.2)	(171.1, 175.9)	1.000
Pak0606	F/6	3/23/06	6/5/06	4	(NA, NA)	(11.2, 35.8)	NA
Pak0607	F/5	3/25/06	6/17/06	21	(3.4, 7.9)	(3, 4.2)	0.744
SI05	F/7	5/11/05	8/25/05	32	(49.6, 98.4)	(105.9, 421.7)	0.912
SJ05	F/8	5/10/05	10/9/05	21	(2.0, 2.0)	(113.5, 437.4)	0.108
SS05	F/6	5/19/05	8/5/05	13	(723.1, 723.1)	(196.8, 1998.9)	0.148
VK05	F/7	5/1/05	7/21/05	32	(5.0, 29.0)	(43.4, 431.8)	0.297
AM06	M/7	4/28/06	11/2/06	87	(3.8, 20.4)	(24.9, 76.4)	0.000
BM06	M/6	5/2/06	6/7/07	44	(38.6, 70.4)	(31.9, 132.9)	0.591
BU08	M/7	5/26/08	10/13/08	46	(4.3, 9.3)	(48.7, 126.7)	0.036
CM06	M/6	5/14/06	1/17/07	60	(8.2, 84.6)	(287.2, 1743.3)	0.001
IB05	M/5	5/16/05	7/9/06	67	(5.6, 15.0)	(21.4, 184.2)	0.020
JJ05	M/7	5/25/05	6/21/06	32	(56.5, 83.4)	(149.0, 946.0)	0.509
JM07	M/3	5/17/07	6/13/07	19	(3.5, 9.3)	(8.7, 10.7)	0.002
JS07	M/4	5/23/07	12/2/07	103	(29.8, 48.1)	(106.5, 1472.3)	0.124
Pak0601	M/6	3/20/06	6/21/06	25	(8.2, 21.2)	(3.1, 17.7)	0.102
Pak0602	M/7	3/21/06	3/23/06	7	(2.7, 4.7)	(NA, NA)	NA
Pak0603	M/5	3/21/06	4/29/06	7	(8.9, 11)	(NA, NA)	NA
Pak0604	M/5	3/21/06	6/25/06	33	(4.3, 22.7)	(24.6, 79)	0.002
SE05	M/pup	5/22/05	6/17/05	21	(1.4, 2.5)	(4.6, 11.5)	0.006
TT05	M/6	5/18/05	2/27/06	55	(42, 862.6)	(6.2, 16.5)	0.033

Sex is abbreviated male (M), female (F). Age is in years. The date of capture and last observation is given for each seal, along with the number of observations. For the ice-bound (December-May) and open-water (June - November) seasons, the mean and maximum distance (km) from breeding site was calculated. A permutation t-test was used to test for a significant seasonal difference in mean distance from breeding site. NAs are given for missing data.

**Figure 1:**
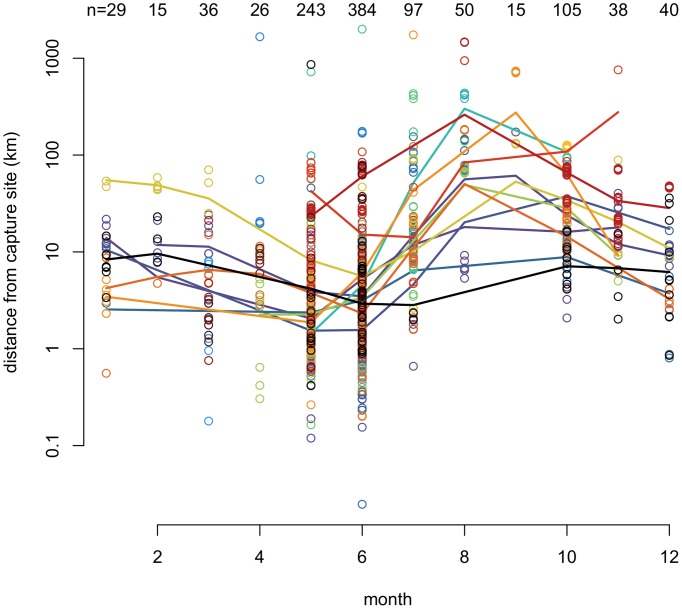
Seasonal localization of ringed seals. The monthly localization of 27 ringed seals measured as the distance from their breeding/capture site. Note, the log_10_ scale of the y-axis. Data are uniquely colored for each seal and a smoothing spline was fit for each individual for which we had at least four months of data. Nine adults were found >400 km from their breeding site between the months of the April and November. In the winter months of December-March, individuals were located within 100 km of their breeding location.

**Figure 2:**
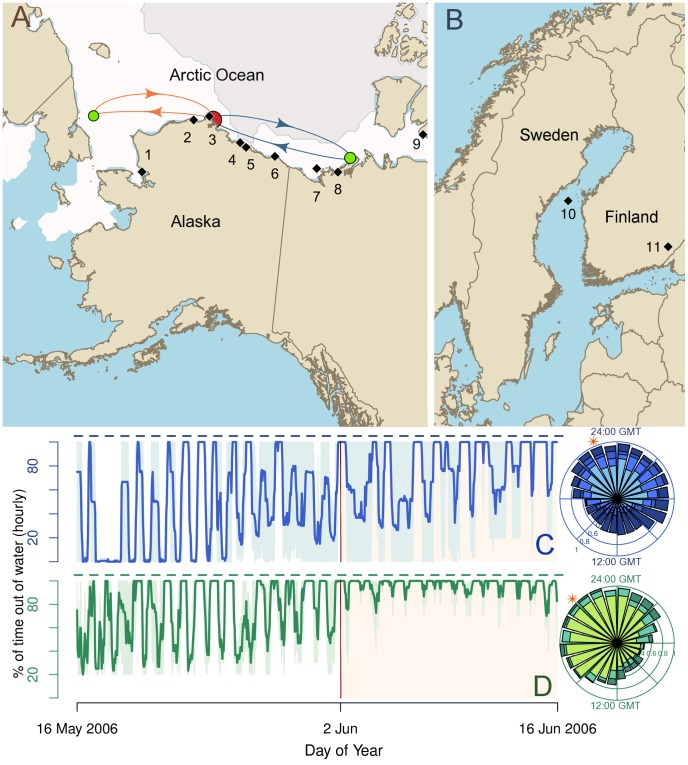
An example of ringed seal migration, sample sites for genetic analysis, and geographic differences in haulout behavior. (A) The black diamonds are the 9 Arctic breeding sites included in our genetic analysis. Red and green circles connected by arrows are movement of an adult male seal tracked using satellite telemetry from May 2005 to May 2006. The red circle indicates his breeding site where he remained during the “*ice-bound*” season when the sea ice extended from the North Pole southward to the oceanic areas colored white. The green circles are locations to which he travelled during the “*open water*” season when the sea ice had retreated north to the region shaded grey. From May - July 2005 he was at his breeding site. He then took a summer trip east (blue arrows) and was located in the Canadian Beaufort in August before returning to his breeding site in October. Upon returning to his breeding site, he embarked upon an autumn trip (orange arrows) west where he was located in November. In May 2006, he was once again located in Barrow. Note, the relative sizes of the circles indicate the number of observations in each region. The breeding sites are in order from west to east: (1) Kotzebue, (2) Peard Bay, (3) Barrow, (4) Oliktok, (5) Prudhoe Bay, and (6) Kaktovik, Alaska; (7) Paktoa, (8) Tuktoyaktuk, and (9) Ulukhaktok/Holman, Canada. (B) The black diamonds numbered 10–11 are the sampling locations in the Baltic Sea and Lake Saimaa, Finland, respectively. (C) Haulout time series and rose diagram of 24-hour haulout cycles for 4 adult seals captured in Peard Bay, Alaska. Haulout time is the percent of the hour the seal was hauled-out atop the sea ice. The dark blue time series is the mean hourly haulout time and the region shaded light blue is the range. The dashed blue lines above the time series indicate the hours from 20∶00 GMT to 08∶00 GMT. Each stacked bar on the rose diagram is the proportion of observations for which a seal was hauled out longer than the mid-range for the day. Each slice represents one of 24 hours of the day, and the lightest bar within a slice is the data for the seal that hauled out the least during that hour; whereas, the darker bars represent seals that hauled out longer during that hour. (D) Haulout time series and rose-diagram for three seals in Paktoa.

Seals tended to move farther from their capture site during June - November, when Arctic sea ice extent is at its annual minimum. With a few exceptions, seals remained closer to their breeding sites during December-May, when ice extent is maximal (Figure 1; ice extent data obtained from the National Snow and Ice Data Center [30]). Of the 24 seals for which we obtained data for both seasons, 10 ranged farther from their breeding sites in June-November (permutation t-test p-values <0.05). One of the ten was a juvenile and another was less than one year old, the remainder were adults. Not all individuals travelled far from their capture site; however, migratory individuals travelled extensively (for an example see Fig. 2a). The seals that travelled extensively moved away from, rather than along, the coast. Seals tagged in Canada were tracked to June at the latest, so observations in July-November were limited to seals tagged in Alaska.

Time spent out of the water by tagged seals provide further insight into their population ecology. Wet/dry sensors on the satellite tags reported the time spent on the ice in May and June 2006. Dry-time data were collected for four seals captured in Peard Bay, Alaska and three seals captured near the Devon Canada drilling site “Paktoa” (N69° 39′ 8.880′′, W136° 29′ 12.128′′) in the southeastern Beaufort Sea. Hereafter, we will refer to the latter site as Paktoa. Despite considerable variation in the time seals spent out of the water (Figure 2c & d), there were distinct patterns within sites. Paktoa seals spent much more time dry than Peard Bay seals. The Paktoa seals always spent at least 12 min of each hour dry, i.e. maximal wet time was 48 min. Whereas, each Peard Bay seal had a maximal wet time of 21–25 consecutive hours, which could be due to flooded lairs. On June 2^nd^, the Paktoa seals reduced the duration of their wet bouts. They shifted from long wet-times that lasted up to 48 minutes in duration, to mean wet-times of less than 12 minutes. This abrupt behavioral change may be due to changes in ice or prey availability. No abrupt behavioral shifts were observed in the Peard Bay seals; however, this could be due to the limited temporal extent of our data. It is also important to note environmental differences between Paktoa and Peard Bay. The water depth at the Paktoa capture sites was 10–13 m; whereas, the depth at the Peard Bay sites was as low as 1.7 m with a maximal depth of 13 m below the ice.

There were also distinct circadian haulout patterns within breeding sites, and these patterns differed between sites (Figure 2c & d). The daily haulout period spanned approximately 15 hours in both Peard Bay and Paktoa. Peard Bay seals, however, hauled out later in the day. They hauled out approximately 4 hours before solar noon and generally finished by 10 hours past solar noon (Figure 2c). In contrast, the Paktoa seals hauled out 8 hours before solar noon and returned to the water by 7 hours past (Figure 2d). We do not know whether these behavioral differences are genetically based or plastic responses to environmental conditions, such as interactions with predators. Polar bears were active at the Paktoa site, which was near their preferred ice-edge habitat. Personnel from an oil-rig at Paktoa regularly observed polar bears (D. Connelly, SSDC, personal communication). At Peard Bay, during the 2006 field season, we identified 43 ringed seal breathing holes, 38 basking holes, and 6 pupping lairs. Seventeen of these (20%) had visible signs of visitation by polar bears and/or Arctic fox. Three basking holes had bear signs and 15 holes had fox signs. We also cannot discount differences in weather between study areas. Typically, basking time increases with radiation and temperatures.

### Variation in Mitochondrial Regions

We collected shed epidermal tissue or biopsies from ringed seals at 11 breeding locations during the breeding season, mid-April to late-May (Figures 2a and 2b). The Arctic subspecies was sampled at nine breeding locations; whereas, the Baltic and Saimaa subspecies were each sampled at a single location. The Cytochrome Oxidase I (COI) region of the mitochondrial genome (mtDNA) was sequenced for 113 individuals from 8 breeding sites: Kotzebue, Peard Bay, and Oliktok Point, Alaska; Paktoa, Tuktoyaktuk, and Ulukhaktok (also referred to by its prior name, Holman), Canada; the Baltic Sea; and Lake Saimaa. Additionally, 99 of these individuals were sequenced at the mtDNA Control Region (CR). The sample sizes for COI and CR are Kotzebue (6, 4), Peard Bay (17, 17), Oliktok (1, 1), Paktoa (14, 14), Tuktoyaktuk (27, 27), Ulukhaktok/Holman (15, 15), Baltic (11, 11), and Lake Saimaa (22, 10).

**Figure 3:**
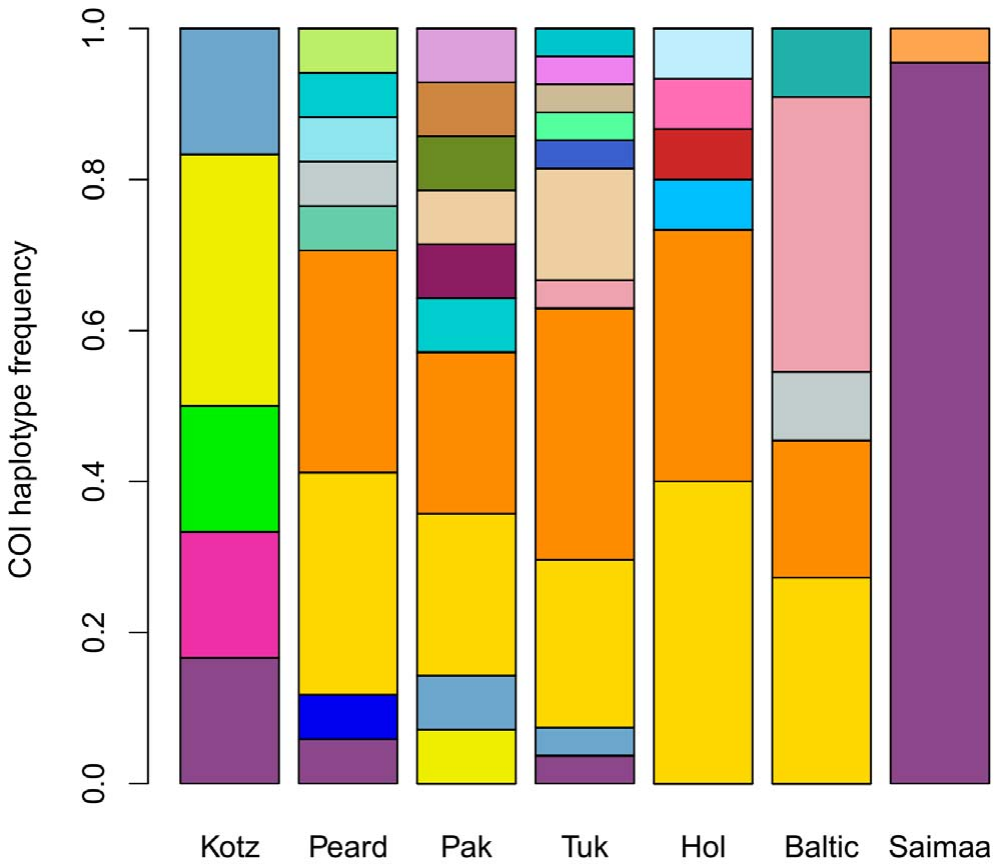
COI haplotype frequencies in 7 populations. The populations are arranged from left to right as follows: Kotzebue (n = 6), Peard Bay (n = 17), Paktoa (n = 14), Tuktoyaktuk (n = 27), Ulukhaktok/Holman (n = 15), Baltic Sea (n = 11), and Lake Saimaa (n = 22). Oliktok was excluded from this figure because we only had one sample from there. Each of the 31 haplotypes is represented by a different color. Lake Saimaa has low haplotype diversity with all but one individual sharing the same haplotype. The Baltic Sea, Ulukhaktok/Holman, Tuktoyaktuk, Paktoa, and Peard Bay all had two prevalent haplotypes (represented by the orange bar and golden bar). Whereas, the haplotypes found in Kotzebue were absent or at low frequency in the other Arctic sites, possibly as an artifact of the low sample size in Kotzebue.

There were 31 unique COI haplotypes among the 113 individuals sequenced at that region. In contrast, all CR haplotypes were unique. Within breeding sites of Arctic and Baltic ringed seals, COI haplotype diversity was high (relative to Saimaa ringed seals), and the dominant haplotypes in the Baltic were also prevalent in Ulukhaktok/Holman, Tuktoyaktuk, Paktoa, and Peard Bay (Figure 3). The Saimaa subspecies was distinguished by low COI haplotype diversity, with all but one of the 22 individuals from Saimaa sharing the same haplotype. Maximum likelihood phylogenies clustered Lake Saimaa individuals into a single clade (Figures S17 and S18), yet there was no phylogeographic signal for Baltic and Arctic ringed seals. We note, however, that the majority of clades in each phylogeny had little bootstrap support. Thus, we relied on additional analyses to determine if there is genetic differentiation among the subspecies and whether migrants are exchanged among them.

### Variation in Nuclear Loci

We analyzed nine nuclear microsatellite loci for 354 individuals from the 11 breeding sites (Figures 2a and 2b), including all individuals from the mtDNA analysis. There was some evidence of departure from Hardy-Weinberg Equilibrium (HWE) within sample sites. Within each population, single locus genotype frequencies were tested for departure from HWE. Following a Bonferroni correction (adjusted α = 0.0005), excess homozygosity was observed 7% of the time, but there was no consistent pattern with regard to which loci had excess homozygosity. We also tested for linkage disequilibrium pairwise between loci within breeding sites. A Bonferroni correction was used (adjusted α = 0.0001), and we found linkage disequilibrium in 4% of the pairwise observations. No two loci, however, were consistently in linkage disequilibrium; therefore, we used all of the data.

**Figure 4:**
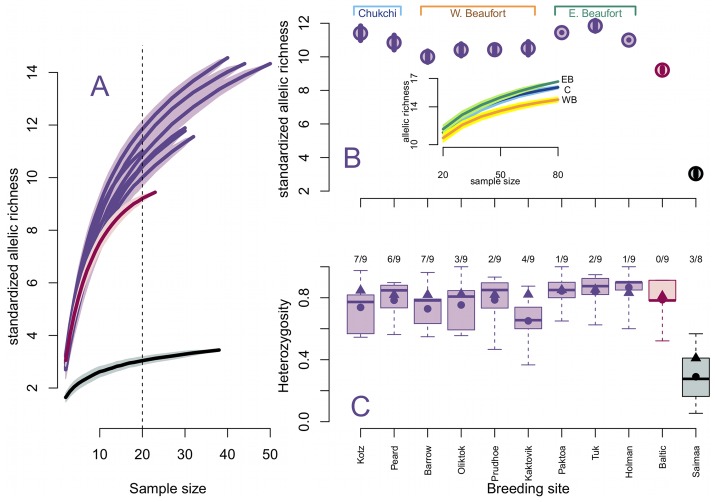
Measures of nuclear genetic variation in Arctic, Baltic, and Saimaa ringed seals. Breeding sites are coded as purple for Arctic ringed seals*;* maroon for Baltic ringed seals**,** and black for Saimaa ringed seals. (A) Relationship between allelic richness (*Â*) and the number of genotypes in a sample ± SD based on 1000 subsampling replicates. Lake Saimaa has low allelic diversity relative to the other subspecies and the Baltic has moderate diversity. (B) Cross sectional data from the standardized allelic richness curve using a sample size of 20 (*Â_N = 20_*). Breeding sites are organized along the x-axis from west to east. Allelic richness is lowest in Lake Saimaa and the Baltic. Within Arctic ringed seals, allelic richness is depressed in the Western Beaufort populations. Inset: allelic richness curves for Arctic ringed seals in the Chukchi Sea region (C), Western Beaufort (WB), and Eastern Beaufort (EB). Even when the genetic variation is pooled for the entire region, it is lower in the Western Beaufort relative to the Chukchi and the Eastern Beaufort. (C) Observed and expected heterozygosity within breeding sites. Box-and-whisker plots represent the observed heterozygosity across polymorphic microsatellite loci with the median represented by the horizontal line. Circles indicate the mean observed heterozygosity across loci and triangles represent the mean expected heterozygosity. The fractions at the top of the plot are the number of polymorphic loci for which the expected and observed heterozygosity are significantly different (p-value <0.05). Despite relatively low allelic richness, the Baltic had relatively high observed and expected heterozygosity, unlike Lake Saimaa, which had both reduced allelic richness and heterozygosity. Note, Ulukhaktok/Holman is denoted as Holman.

The estimated mean number of alleles per locus was used as our measure of allelic richness, a proxy for genetic diversity within each sample site. Due to the variation in sample size among sample sites, we look at the relationship between sample size, *N*, and sample-size-standardized allelic richness, *Â_N_* (Figure 4a). The allelic richness standardized to the smallest sample size (*Â_N = 20_*), was used to compare genetic diversity among populations and was found to be lower in the Baltic and Lake Saimaa subspecies than in the Arctic (Figure 4b). The standardized allelic richness (*Â_N = 20_*) is significantly lower in the Baltic than any of the Arctic populations*;* and lower in Lake Saimaa relative to the Baltic (p-values <2.2e-16); with the Baltic containing three times more allelic richness than Saimaa.

In addition to the population-level analysis, we investigated the regional differences within Arctic ringed seals by pooling the Arctic breeding sites into three geographic units: Chukchi Sea, the Western Beaufort Sea, and the Eastern Beaufort Sea. Allelic richness was higher in the Eastern Beaufort (*i.e.* Tuktoyaktuk, Paktoa, and Ulukhaktok/Holman) and Chukchi Sea populations (Kotzebue and Peard Bay) and was depressed in the Western Beaufort Sea (Barrow, Oliktok, Prudhoe Bay, and Kaktovik). The Western Beaufort region has 1–4 fewer alleles at 5 of the 9 loci, significantly reducing its allelic richness (p-value <2.2e-16; Figure 4b inset). Reduced allelic richness in the Western Beaufort may be indicative of low genetic variation within the region. The presence of null alleles in the Western Beaufort, however, might also explain the reduced allelic richness.

Mean observed heterozygosity (*H_o_*) was less than expected heterozygosity (*H_e_*) at all sites except Ulukhaktok/Holman, where locus-specific *H_e_* and *H_o_* were not significantly different at eight of the nine loci (Figure 4c). The sites with the lowest *H_o_* were Lake Saimaa, Kotzebue, Barrow, Oliktok, and Kaktovik; the latter three being part of the Western Beaufort. Despite low allelic richness, the Baltic had relatively high *H_o_* and *H_e_*, unlike Lake Saimaa, which had both reduced allelic richness and heterozygosity. We measured heterozygosity for each locus independently to check for potential bias in heterozygosity estimates due to null alleles (Figures S19 and S20). Lake Saimaa had the lowest observed heterozygosity for each locus. The difference between *H_e_* and *H_o_* was particularly punctuated at locus SGPV16. Thus, we measured heterozygosity with this locus excluded and found that the Eastern Beaufort continued to have higher mean *H_o_* than the Western Beaufort and Chukchi sites (Figure S21). With SGPV16 removed, Kaktovik still had lower mean *H_o_* than all other Arctic sites and the Baltic. The pattern of elevated heterozygosity in the Eastern Beaufort and Baltic, relative to the Chukchi and Western Beaufort, was not only robust to the removal of SGPV16 from the analysis, but also additional loci (S22–S25). Due to the use of shed-epidermis as the primary source of DNA from the Chukchi and Western Beaufort, there is the potential influence of sample quality on the levels of diversity observed. Swanson et al. [25] demonstrated that shed-skin yields lower DNA quantity and purity than tissue samples taken from captured animals. There is no significant difference in heterozygosity, however, based on sample type (shed-skin vs. tissue collected as biopsies) [25]. The DNA we extracted from shed-skin collected in the Chukchi and Western Beaufort had the same level of purity as the samples used in the Swanson et al. study (Figure S26).

**Figure 5:**
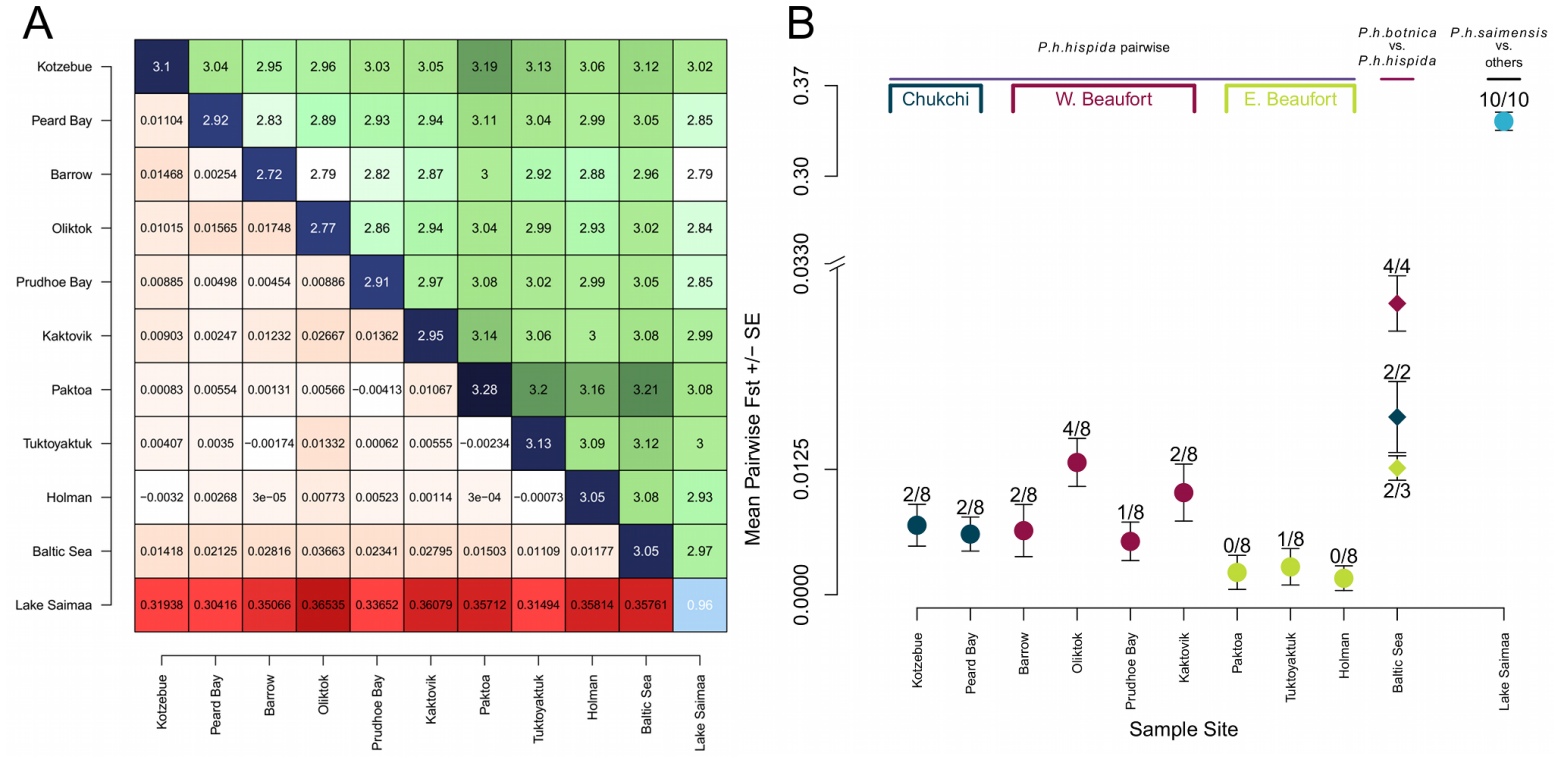
Population differentiation based of nuclear microsatellites: pairwise differences among populations and pairwise F_ST_. (A) Pairwise F_ST_ (below diagonal), average number of pairwise differences within populations (diagonal), and average number of pairwise differences between populations (above diagonal). Color intensity indicates the relative magnitude of the values. (B) Pairwise fixation indices (F_ST_) between subspecies and among breeding populations of Arctic ringed seals. Populations are arranged across the x-axis from west to east. Blue, maroon, and green circles are mean pairwise F_ST_ ± SE values between the population labeled and the other 8 Arctic populations. The blue, maroon, and green diamonds represent the mean pairwise F_ST_ between the Baltic subspecies and the Chukchi Sea populations, Western Beaufort populations, and the Eastern Beaufort populations of the Arctic subspecies, respectively. The light blue circle is the mean F_ST_ taken pairwise between the Lake Saimaa and Baltic subspecies along with each pairwise F_ST_ between Lake Saimaa ringed seals and the nine Arctic breeding sites. The labels near each point represent the fraction of pairwise comparisons for which the pairwise F_ST_ was significantly different from zero (p-value <0.05). The Baltic is more similar to all the Eastern Beaufort breeding sites than several of the Arctic sites are to each other. Although the Baltic and Eastern Beaufort we not highly divergent, the mean F_ST_ for the Baltic and the Western Beaufort can be interpreted as moderate differentiation. Lake Saimaa ringed seals are genetically highly divergent from the other seal populations. Note, Ulukhaktok/Holman is denoted as Holman.

We also estimated the amount of genetic differentiation between breeding sites using pairwise fixation indices (F_ST_). F_ST_ for Saimaa pairwise with the Baltic and the nine Arctic breeding sites ranged from 0.30–0.37, where F_ST_ values >0.25 are generally taken to represent pronounced levels of genetic differentiation (Figure 5) [9]. In contrast, when the Baltic was compared to the Arctic, F_ST_ values were low (range 0.011–0.037). F_ST_ for the Baltic and Ulukhaktok/Holman was not significantly different from zero (p-value >0.05), and the Baltic was more similar to all the Eastern Beaufort breeding sites than several of the Arctic sites were to each other. Although the Baltic and Eastern Beaufort were not highly divergent, the mean F_ST_ for the Baltic and the Western Beaufort was 0.029±0.0029, which could be interpreted as moderate differentiation (Figure 5). Within the Arctic, pairwise differences between the Eastern Beaufort and other sites were not significantly different from zero, with the exception of Tuktoyaktuk pairwise with Oliktok, which had an F_ST_ of 0.013. In contrast to the Eastern Beaufort, the Chukchi and Western Beaufort had higher mean F_ST_ with Oliktok being more divergent than the other sites.

### Genetic Variation, Panmixia, & Gene Flow

We quantified the genetic differences among and within breeding sites, with and without the inclusion of Lake Saimaa, using Analysis of Molecular Variance (AMOVA). The majority of genetic variation was found within populations rather than among populations. With the inclusion of seals of Lake Saimaa (AMOVA I), significant levels of genetic variance were attributable to both among- and within-site differences (p-values <0.05; Table 2). Genetic variance attributed to differences among sites was 19.56%, 14.02%, and 7.51% for COI, CR, and microsatellites, respectively. When we excluded Lake Saimaa (AMOVA II), however, among-site variance fell to 1.18%, 2.78%, and 0.86% (same order as above), and the amount of variance among sites was no longer significant for COI (p-value = 0.239). Taken together, the AMOVAs revealed that over 97% of the observed genetic variation in the Arctic and Baltic is harbored within breeding sites rather than between sites. Furthermore, due to the low genetic diversity of the Saimaa subspecies, among-site genetic differences were elevated when *P. h. saimensis* was included in AMOVAs but remained far below the within-site variance (Table 2; pairwise matrices in File S1).

**Table 2:** Analysis of molecular variance (AMOVA) based on Cytochrome Oxidase I (COI), the Control Region (CR), and 9 microsatellite loci. AMOVA I contains Arctic, Baltic, and Lake Saimaa subspecies; AMOVA II excludes the Lake Saimaa subspecies.

	AMOVA I			AMOVA II		
	COI	CR	Microsatellites	COI	CR	Microsatellites
Variance component	% variance			% variance		
Among sites	19.56	14.02	7.51	1.18	2.78	0.86
Within sites	80.44	85.98	92.49	98.82	97.22	99.14
p-value^a^	0.000	0.000	0.000	0.239	0.048	0.000

a p-value obtained from significance test (16000 permutation); P(random value>observed value of variation among sites).

**Figure 6:**
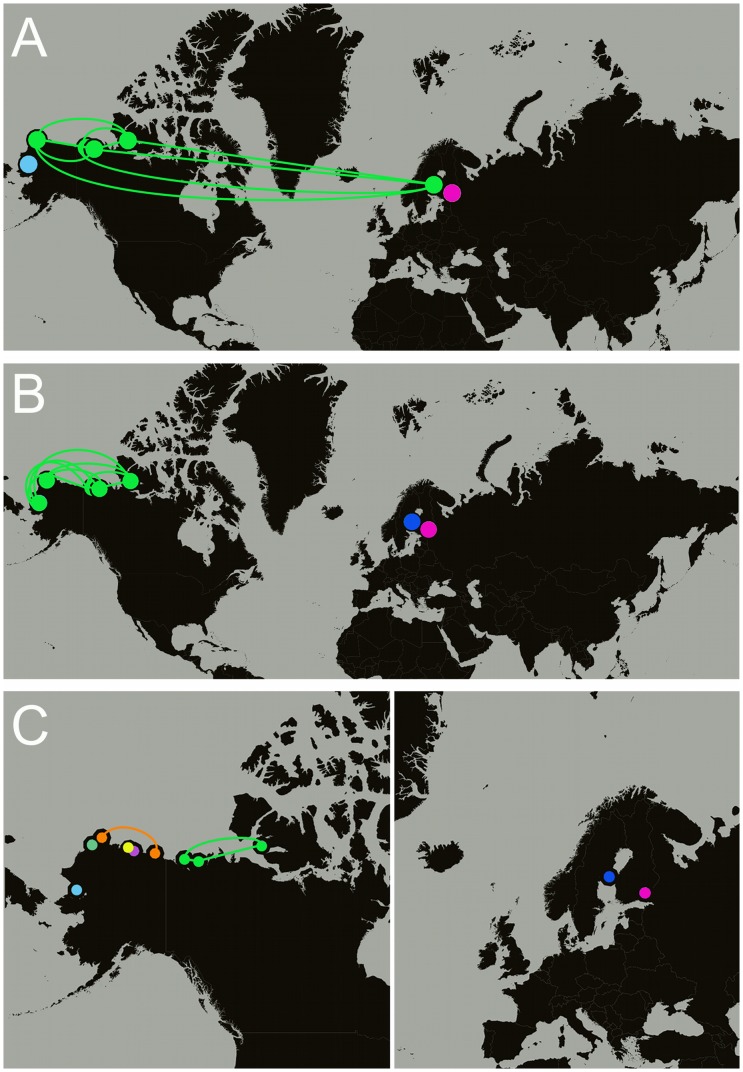
Panmixia and genetic differentiation between subspecies and breeding populations of ringed seals. Breeding sites from left-to-right: Kotzebue, Peard Bay, Paktoa, Tuktoyaktuk, Ulukhaktok/Holman, Baltic Sea, and Lake Saimaa. Populations with the same color and connected by a line were deemed panmictic based on pairwise permutation tests using (A) mtDNA Cytochrome Oxidase I, (B) mtDNA control region, and (C) microsatellites. Non-panmictic sites are significantly differentiated from other sites (p-values <0.05). Breeding sites left-to-right in panel C: Kotzebue, Peard Bay, Barrow, Oliktok, Prudhoe, Kaktovik, Paktoa, Tuktoyaktuk, Ulukhaktok/Holman, Baltic Sea, and Lake Saimaa.

The AMOVAs demonstrated little genetic variation among breeding sites, suggesting interbreeding across sites. In order to determine whether any of our sites (taken pairwise) are panmictic, we employed a nonparametric method of testing a null hypothesis of panmixia vs. genetic differentiation for pairs of sample sites. The statistical test, permtest, based on the work of Hudson, Boos, and Kaplan [31] was preformed using each of our genetic markers independently (i.e. microsatellites, COI, and CR). All three markers signaled that Lake Saimaa is genetically differentiated from all other sample sites (p-values <0.003). All permutation procedures also showed Paktoa, Tuktoyaktuk, and Ulukhaktok/Holman (the three Easternmost *P. h. hispida* breeding sites) to be panmictic (p-values >0.05). The CR and the microsatellites suggest that the Baltic Sea is also genetically differentiated (p-values <0.05); however, COI suggests that that the Baltic is panmictic with Ulukhaktok/Holman, Tuktoyaktuk, Paktoa, and Peard Bay (p-values >0.05; Figure 6).

**Figure 7:**
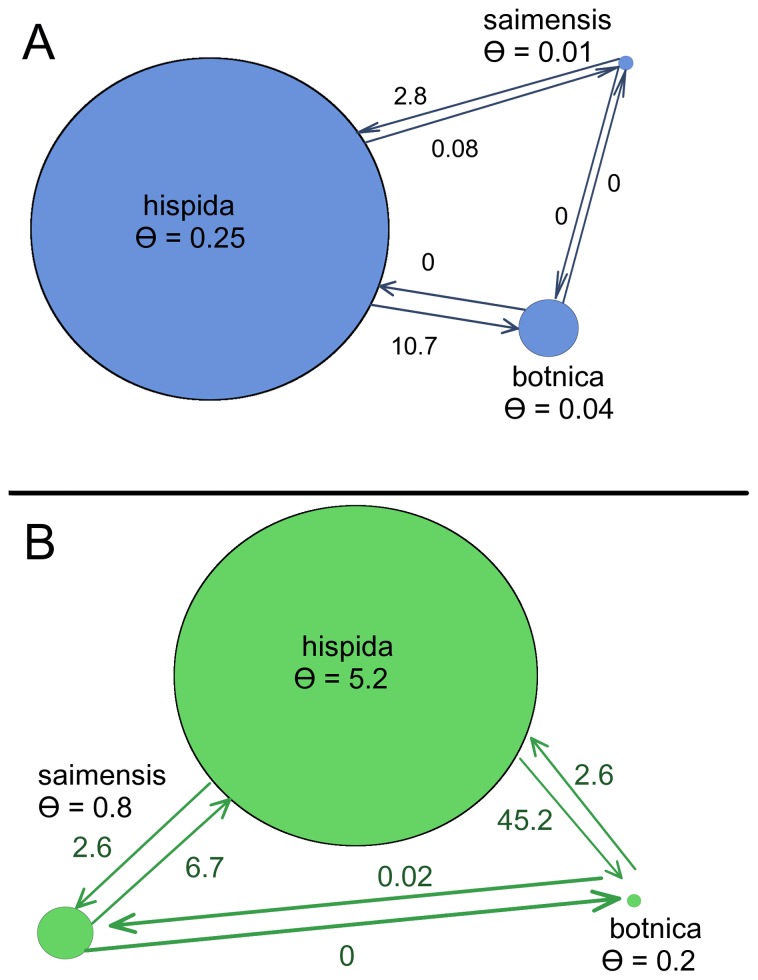
Maximum likelihood parameter estimates of mutation-rate-scaled effective population sizes and migration rates. (A) Mutation-scaled effective population size (Θ) estimates based on mtDNA. Each circle represents a ringed seal subspecies and the relative size of the circle is indicative of the effective population size. Arrows are labeled with the estimated number of migrants per generation. (B) Estimates based on nuclear microsatellites.

We estimated the historical and contemporary migration rates among all three subspecies using the maximum likelihood parameter estimation procedure in the program MIGRATE [32]–[34] to determine whether there is ongoing gene flow between the Baltic and the Arctic. Historical migration rates were estimated using COI and CR, whereas migration rates based on the microsatellite data are assumed to be reflective of contemporary gene flow. The maximum likelihood parameter estimates of historical migration from the Arctic to the Baltic and Saimaa are 10.7 and 0.08 migrants per generation, respectively. The contemporary estimates are 45.2 and 2.6 migrants per generation, respectively (Table 3 and Figure 7). In contrast, the migration from the Baltic to the other subspecies was zero migrants per generation historically; and contemporary estimates are 2.6 migrants per generation to the Arctic and 0.02 to Lake Saimaa. Lastly, movement from Lake Saimaa to the Baltic was inferred to be zero both historically and contemporarily; whereas, migrants per generation from Lake Saimaa to the Arctic were 2.8 historically, and are 6.7 currently. With regard to migration between Lake Saimaa and the Arctic, the high levels of diversity in the Arctic, contrast with low levels in Saimaa, and the time since isolation of the two, may be driving unlikely migration rate estimates.

**Table 3:** Maximum likelihood estimates of migration parameters for each subspecies.

Region	Receiver Subspecies	Ln(L)	θ [xNμ]	M from *hispida*[m/μ]	M from *botnica*[m/μ]	M from *saimensis*[m/μ]
mtDNA	*hispida*	−69.022	0.24577	–	0.0000	11.3677
	*botnica*	−69.022	0.03669	290.9826	–	0.0000
	*saimensis*	−69.022	0.00986	7.8192	0.0000	–
microsatellite	*hispida*	−107.963	5.23813	–	0.4897	1.2845
	*botnica*	−107.963	0.20831	216.7864	–	0.0000
	*saimensis*	−107.963	0.84026	3.0435	0.0182	–

We also estimated the mutation-scaled effective population size (Θ) for each subspecies. Based on the mtDNA data, the effective population size of the Arctic subspecies (Θ = 0.25) is 6× larger than that of the Baltic subspecies (Θ = 0.04); and the effective population size of Baltic subspecies is 4× that of the Saimaa subspecies (Θ = 0.01) (Table 3). The microsatellite data also suggested a large effective population size for the Arctic subspecies (Θ = 5.24); however, the estimate for Lake Saimaa (Θ = 0.8) was larger than that of the Baltic (Θ = 0.2). The MIGRATE analysis provided support for there being gene flow between the Baltic and the Arctic, in contrast to the relative isolation of Lake Saimaa. The model used to estimate the migration rate parameters and effective population sizes, however, assumes equilibrium gene flow, which is an assumption unlikely to be met by our subspecies. Thus, the absolute numbers may not be representative of the realized number of migrants between the subspecies in recent generations. The high amount of gene flow between the Arctic and the Baltic, as indicated by the MIGRATE analysis, however, is corroborated the low levels of genetic divergence between the two and the ability of ringed seals to seasonally travel long distances. Refer to File S3 for a summary of profile likelihood percentiles for all parameters estimated using MIGRATE.

## Discussion

We used behavioral and genetic data to determine the potential for, and realized amount of, gene flow among subspecies and populations of ringed seals. While 88% of seals (23 of 26) remained within 100 km of their breeding sites during the winter and spring months, 60% of the tracked seals (15 of 25) were observed a hundred to over 1,000 km away from their breeding site during the summer months when food is abundant, ice cover is minimal, and Arctic waters can be navigated freely. Our observations of seal locations were numerous in spring and early summer and sparse the remainder of the year due to limitations of the tags. Nonetheless, the observed movements demonstrated that ringed seals can migrate >1000 km within the span of several months (Figure S27). Thus, ringed seals have high dispersal capabilities, a precursor for gene flow.

Our movement results are concordant with recent work by Harwood et al. 2012 [35] and Crawford et al. 2012 [36], who investigated the movements of ringed seals tagged in Western Canada and Kotzebue Alaska, respectively. The seals in the Western Canada study all displayed a similar migratory behavior; following their release in the Canadian Beaufort Sea in September, they travelled west, offshore of the North Slope of Alaska, and into the Chukchi Sea. Several of these seals were located in Russian coastal waters between the months of October and December, and one individual moved south into the Bering Sea [35]. Each of those seals, with the exception of a pup, travelled 700–4600 km. Similarly, the seals in the Northern Alaska study [36] displayed extensive movement in the Chukchi and Bering Seas with strong seasonality in their rate of travel. The rate and directionality of the movement observed was tightly linked to sea ice conditions, and travel rates were least from January-March.

Our haulout results demonstrate that the use of sea ice can vary greatly on time scales as short as a month. Haulout behavior impacts migration because dry-times place an upper bound on the extent of migration. A promising area for future research is coupling seasonal haulout time-series with long-term tracking data to understand how migratory ringed seal use the ice habitat during migration and the time spent in/out of the water may inform the estimation of swimming speeds.

We found low to moderate genetic differentiation between Baltic and Arctic ringed seals. The mtDNA-based phylogenies (Figures S17 and S18), microsatellite-derived fixation indices (Figure 5), and AMOVAs (Table 2) each suggested little genetic differentiation between *P. h. botnica* and *P. h. hispida* even though these subspecies were thought to be effectively geographically isolated for thousands of years [21]. Our results were in keeping with those of Palo et al. [21], [22] and Davis et al. [23]. Palo et al. estimated that there are nine effective immigrants per generation from the Arctic to the Baltic, and we estimated 10.7 based on mtDNA and 45.2 based on microsatellites.

In the early part of the 20^th^ century, harvests reduced the population of the Baltic ringed seal from ∼200,000 individuals to ∼5,000 [37]. Despite that recent bottleneck, the similarity in the genetic composition of Baltic and Arctic ringed seals was unexpected. In the face of seemingly strong geographic barriers, there seems to be effective dispersal into the Baltic from the Arctic. Previous studies have shown that as few as 10 migrants per generation are enough to prevent populations from undergoing genetic differentiation due to genetic drift [38]. Thus, our mtDNA-based estimate of 10.7 and microsatellite-based estimate of 45.2 migrants per generation into the Baltic from the Arctic are sufficient for *P. h. botnica* to maintain high genetic diversity. Our tracking study shows ringed seals have the physical capability of migrating on a pan-Arctic scale, and other telemetry studies have demonstrated ringed seals can navigate narrow waterways and fjords [39]. Thus, it is plausible that ringed seals from the Beaufort, Greenland, and Barents Seas traverse the Norwegian and North Sea to immigrate into the Baltic.

Immigration from the Arctic into the Baltic Sea has potentially countered the effects of genetic drift. Nevertheless, there are physical differences between Baltic and Arctic ringed seals that were hitherto considered evidence against contemporary gene flow [40]. The most notable difference between the subspecies is darker pelage in the Baltic seals. Our study focused on neutral loci, rather than those involved in pelage characteristics, thus we cannot address this particular characteristic. It is feasible, however, that the lack of genetic differentiation we have found in this study is not reflected in genes for adaptive traits such as pelage color. The connectedness between the Arctic and Baltic is a particularly telling feature of ringed seal ecology, the importance of which has been underappreciated in management strategies. The biological relevance of gene flow from Arctic to Baltic ringed seal populations should not be ignored because of phenotypic differences between the subspecies. The geographic scale at which migrants can be exchanged is circumpolar, and immigration into the Baltic Sea may contribute to the persistence of the Baltic subspecies by protecting against diminished genetic variation, inbreeding depression, and effects of genetic drift from bottlenecks.

In contrast to the high level of connectedness between the Arctic and Baltic ringed seals, the Lake Saimaa subspecies is highly differentiated from the others and is characterized by low genetic diversity. We found Lake Saimaa to have depressed haplotype diversity (Figure 3), low allelic richness and heterozygosity (Figure 4), and high fixation indices (Figure 5); also, Saimaa seals were consistently genetically distinct from the other subspecies (Figure 6). This echoes the findings of Palo et al. who also found a reduction in microsatellite diversity in the Saimaa ringed seal compared to the Baltic and Arctic [22]. Our results are also in keeping with recent work by Valtonen et al. [41] who found the variability in the mtDNA control region to be substantially lower in Lake Saimaa relative to the Baltic and the Lake Ladoga subspecies (*P. h. ladogensis*). They also found the differentiation between the Baltic and Lake Ladoga to be much lower (F_ST_ = 0.028) than that of Saimaa taken pairwise with the other two species (F_ST_ >0.227), perhaps due to a river connection between Ladoga and the Baltic. Like its Baltic counterpart, the Saimaa ringed seal has been severely reduced through harvests, drowning in fishing gear, lowered water levels, and DDT and PCB contamination [42]. Considering its history and small current census size (N <300), genetic drift likely explains the differentiation of the Saimaa ringed seal from the other subspecies.

The genetic differentiation of Lake Saimaa from the other two subspecies and the genetic similarity between the Arctic and the Baltic give weight to the conclusion drawn by Berta and Churchill [43] who reviewed morphological and genetic studies of ringed seals and concluded that the Baltic ringed seal should not be recognized as a valid subspecies due to their lack of differentiation from the Arctic ringed seal; whereas, the Saimaa ringed seal can be considered a subspecies based on morphometrics. In addition to the gene flow observed between Arctic and Baltic ringed seals, our data indicate that there is gene flow among subpopulations of the Arctic subspecies. Similar to Davis et al., our estimates of the amount of genetic differentiation among Arctic subpopulations suggests little regional differentiation within the subspecies (Figure 5), and panmixia may be found among Arctic breeding sites (Figure 6).

There are three particularly striking characteristics of the Western Beaufort breeding sites (Barrow, Oliktok, Prudhoe Bay, and Kaktovik) of the Arctic subspecies. First, the Western Beaufort sites had the lowest allelic richness within the subspecies, both when taken individually and when aggregated at a regional level. Secondly, Kaktovik also had much lower levels of heterozygosity than the Baltic subspecies and other Arctic breeding sites. Lastly, the mean F_ST_ for Oliktok and Kaktovik, taken pairwise with other Arctic breeding sites, was elevated to a level that is comparable to the amount of differentiation between the Baltic and the Arctic. The apparent diminished genetic variation in the Western Beaufort Sea suggests that ringed seals in this region may be more vulnerable to population declines.

## Materials and Methods

### Ethics Statement

Marine Mammal Protection Act scientific research permits were obtained from the United States National Marine Fisheries Service Office of Protected Resources (Scientific Research permit Numbers: 350-1739-00, 782-1694-00), and the University of Alaska Fairbanks Institutional Animal Care & Use Committee (IACUC) approved animal-handling protocol titled: “Population Genetics of Ringed Seals”, protocol number 08–11. Research conducted in the Canadian Arctic under Scientific License issued by the Department of Fisheries and Oceans (DFO), Canada (license numbers SLE-04/05-328 and SLE–05/06-322). Animal Care Use Protocol was also approved by DFO (protocol number UFWI-ACC-2004-2005-001U). Baltic ringed seal tissue samples were collected from animals harvested for scientific purposes by Finnish Game and Fisheries Research Institute (FGFRI) under special permission from the Finnish Ministry of Agriculture and Forestry. The special permission allowed FGFRI to sample Baltic ringed seals in April 2007 and 2008 (annual harvest of 10–15 individuals). Saimaa ringed seal tissue samples collected by FGFRI were from seals that were fisheries by-catch or found stranded.

### Collection of Behavioral Data

Seals were live-captured at breeding sites in Peard Bay, Alaska (n = 15); Paktoa, Canada (n = 4); Barrow, Alaska (n = 2); and Kotzebue, Alaska (n = 2). See [44] for capture protocol. Seals were tagged on the hind flipper with Wildlife Computer’s Smart Position and Temperature (SPOT) satellite transmitters. The SPOT tags provided location and haulout information. Data were transmitted to satellites on intermittent days if the tag’s conductivity switch indicated the seal was at the surface or out of the water. Haulout data were transmitted as hourly values of dry time (percent of each hour the wet/dry sensor reads dry), which we interpreted as the time spent out of the water. All animals were tracked using the Argos satellite system. Data were downloaded by the United States National Marine Mammal Laboratory and processed for quality. The R package ‘argosfilter’ [45] was used to filter out low quality and/or unrealistic observations. Locations requiring unrealistic swimming speeds (>2 m/s) were removed. The statistical program R was used for statistical analysis of location and haulout data. Maps were made using R and the open source map software TileMill.

### Collection & Analysis of Genetic Data

#### Sample collection & DNA extraction

The majority of samples used in this study consisted of epidermal tissue that seals shed on the ice surrounding their breathing holes. Molted epidermis was collected from seals in Kotzebue Sound, Peard Bay, Point Barrow, Oliktok Point, Prudhoe Bay, and Kaktovik (Figure 2a). In addition to molted epidermis, we collected biopsies from the hind flippers of seals live-captured for our telemetry study. Furthermore, DFO provided biopsies from individuals captured or harvested during the breeding season in the western Canadian Arctic near Paktoa, Tuktoyaktuk, and Ulukhaktok/Holman (Figure 2a). Biopsies from the Baltic and Finnish subspecies were provided by FGFRI (Figure 2b). Following collection, tissue samples were dried and then subsequently kept frozen at −80°C until DNA was extracted. The majority of DNA was extracted using a QIAGEN Dneasy kit (Qiagen, Valencia, CA); all other samples were processed using proteinase K and ammonium acetate according to a Puregene DNA isolation protocol (Gentra Systems, Minneapolis, Minnesota).

#### Mitochondrial DNA sequencing

Polymerase chain reaction (PCR) was used to amplify mitochondrial DNA. Using a *P. h. hispida* mtDNA sequence obtained from GenBank, the program Primer3 [46] was used to develop primers for the Cytochrome Oxidase I (COI) region and Control Region (CR). Mitochondrial DNA amplification consisted of an initial denaturation step for 6 min, at 94°C, followed by 48°C for 1 min, 72°C for 1 min 30 s, 34 cycles of 1 min at 94°C, 5 s at 72°C, and refrigeration at 4°C using the *P. hispida* COI left primer 5′-TTA ATC CGC GCA GAA CTA GG-3′ and right primer 5′- GCA GGG TCG AAG AAT GTT GT-3′ (sequence size = 640 bp); and the CR left primer 5′- GTA AAC AAC CCC ACC ACC AG-3′ and right primer 5′- CGC CTC ATG GTT GTA TGA TG-3′ (sequence size = 1454 bp). The PCR products and primers were shipped to the High-Throughput Genomics Unit, Department of Genome Sciences, University of Washington, for purification and cycle sequencing. Cycle sequencing was done in both the forward and reverse direction. Thus, two independent, but complimentary, sequences were supplied for both the COI region and CR of each individual. To check the precision of the High-Throughput Genomics Unit, two individuals were repeatedly sequenced independently.

A total of 113 individuals from 8 breeding sites had their mitochondrial DNA COI region sequenced, and a subset of these were also sequenced at CR (Kotzebue Sound, n = 6; Peard Bay, n = 17; Oliktok Point, n = 1; Paktoa, n = 14; Tuktoyaktuk, n = 27; Ulukhaktok/Holman, n = 15; Bothnia Bay, n = 11; Lake Saimaa, n = 22). Note, mitochondrial DNA was not sequenced from Barrow, Prudhoe Bay, or Kaktovik due to the low quantity of shed epidermis collected for each animal or sample degradation. The bioinformatics software Geneious [47] was used for editing sequences and running ClustalW sequence alignments. Complimentary sequences were used together for optimal editing. Sequence quality varied greatly between individuals. The greatest uncertainty in sequence accuracy occurred at the ends of each sequence; thus, we decided to work with a 359 base pair (bp) subset (bps 90–564) of the COI region that had high quality sequences across all samples and a 476 bp subset (bps 90–564) of CR.

Unique haplotypes were identified using the program Arlequin 3.5.1.3 [48]. The program PhyML 3.0 [49] was used to infer phylogenies. Before building the phylogenies, the program FindModel was used to identify an appropriate model of nucleotide substitution for each mtDNA region. The TN93 nucleotide substitution model was used for the CR phylogeny along with a discrete gamma model with 4 rate categories. The GTR nucleotide substitution model was used for COI with a discrete gamma model with 6 rate categories. For each region, PhyML estimated the gamma shape parameter, along with the proportion of invariable sites and 1000 bootstrap data sets were used to measure the support for each clade.

#### Nuclear DNA genotyping

A total of 354 samples were amplified at 9 microsatellite loci: SGPV9, SGPV10, SGPV11, SGPV16, Hg 4.2, Hg 6.1, Hg 6.3, Hg 8.10, Hl-16 [50]–[52]. Reverse primers were labeled on the 5′ end with a fluorescent dye (FAM, TET, or HEX). Microsatellite amplification was conducted on an Eppendorf MasterGradient Thermocycler (Brinkman Instruments Inc., Westbury, NY, USA) and consisted of an initial denaturation step for 2 min at 94°C followed by three cycles of 20 s at 94°C, 20 s at 53–55°C, and 5 s at 72°C. This was followed by 33 cycles of 15 s at 94°C, 20 s at 53–55°C, 10 s at 72°C, and a terminal extension step of 3 min at 72°C [25], [52]. The PCR products were run through an ABI Prism 310 Genetic Analyzer using GENESCAN analysis 3.1.2 and GENOTYPER 2.5 software (Applied Biosystems, Foster City, CA, USA) to determine genotypes.

Genotypes were examined for null alleles, consistent repeat motifs, allelic dropouts, and calling errors by MicroChecker [53]. The program GENECAP [48] was used to determine if shed skin samples were from recaptured individuals. We used a one mis-match model, which compared all genotypes in the data set to determine which samples differed by either zero or one allele. Individuals flagged by GENECAP were considered duplicate genotypes; we retained only one genotype from each individual for analysis. All genotypes were then analyzed using Arlequin 3.5.1.3 [48], GENEPOP [54], and GenAlEx [55] to check for deviations from Hardy-Weinberg equilibrium and linkage disequilibrium. Arlequin was also used to calculate the F-statistic F_ST_, average pairwise differences within and between populations, and measures of heterozygosity [56], [57]. The R package standArich, developed by F. Alberto [58], was used to estimate population allelic richness standardized to sample size.

#### Measuring genetic variation, panmixia, & gene flow

The program Arlequin 3.5.1.3 [48] was used for analysis of molecular variance (AMOVA) and measuring genetic distance among individuals. Standard AMOVAs were run and significance testing of AMOVA indices was done using the permutation procedure (n = 16000 permutations). Distance matrices for the mtDNA regions were computed using a Tamura-Nei model with a γ parameter of 0.251 for COI and 0.164 for CR. The distance matrices for the microsatellite AMOVAs were computed based on the number of different alleles.

The program permtest [31] (distributed by Richard Hudson of the University of Chicago) was used to test for geographical subdivision among sample sites. Permtest, based on the work of Hudson, Boos, and Kaplan, implements a nonparametric method of testing a null hypothesis of panmixia vs. genetic differentiation among sample sites. Taking two samples sites at a time, permtest calculates K_i_, the average genetic distance between individuals of sample site i, where i = 1, 2. The sample size weighted average of K_i_ is defined as the within-site genetic distance between individuals, and is denoted K_S_; and K_T_ is defined as the mean genetic distance between individuals, regardless of the sample site from which they were drawn. The test statistic (K_ST_), defined as 1- (K_S_/K_T_), estimates the level of genetic differentiation between sample sites, and uses a permutation procedure to determine whether the observed value of K_ST_ is statistically significant. Tests for panmixia were run independently using CR, COI, and the microsatellites. The input data for the analyses were genetic distance matrices containing pairwise measures between individuals.

For the permtest analysis, mtDNA genetic distances were calculated using the program MEGA (Molecular Evolutionary Genetics Analysis version 5.0) [59]. The nucleotide substitution model used was the Tamura-Nei+γ model with α = 0.25103 for COI and α = 0.164 for CR (K = 4). GenAlEx [55] was used to calculate nuclear genetic distances between individuals based on their nine-locus genotypes. Taking two individuals at a time, and arbitrary alleles *i*, *j*, *k*, and *l*, the single-locus genetic distance is 0 for genotype pair (*ii,ii*) or (*ij,ij*), 1 for (*ii,ij*) or (*ij, ik*), 2 for (*ij,kl*), 3 for (*ii, jk*), and 4 for (*ii,jj*). The single-locus genetic distances were then summed to obtain the overall distance. The resulting genetic distances were used in permtest to test for panmixia pairwise between the sample sites for which we had >1 individual. For each test 5000 permutations were used for significance testing.

The program MIGRATE 3.3.2 [32]–[34] was used to estimate the mutation-scaled effective population sizes (Θ) and migration rates (M) for the three subspecies using two datasets independently: mtDNA and microsatellites. For each data set, a multi-phase inference procedure was implemented, with 9–10 phases. In the first phase, the starting estimates for Θ and M were based off of F_ST_ values. Each subsequent phase used estimates from previous phases. For each estimation phase, the maximum likelihood search strategy was utilized to estimate the full migration model (i.e. all pairwise bidirectional migration rates) using anywhere from 1–5 runs of MIGRATE. For each run, the number of short chains was 10 and the number of long chains was 3, with the burn-in for each chain being 10000. The number of recorded genealogies in short chains ranged from 500 to 1000, and the number of recorded genealogies in long chains was always 10× that of short chains. The short and long sampling increments were set equal to each other, but they differed between phases and the values ranged from 20–100. The maximum likelihood estimates provided in our results are those estimates with the highest log likelihood of all of the phases.

## Supporting Information

Figure S1 Movement of satellite-tracked ringed seals. Each maps shows the locations for a single individual (seal name given in bottom right corner). Each individual’s capture site is marked with a star and locations triangulated by satellite are color-coded based on the month. Insets are provided to show the general location of the sites.
(TIFF)

Figure S2 Movement of satellite-tracked ringed seals. Each maps shows the locations for a single individual (seal name given in bottom right corner). Each individual’s capture site is marked with a star and locations triangulated by satellite are color-coded based on the month. Insets are provided to show the general location of the sites.
(TIFF)

Figure S3 Movement of satellite-tracked ringed seals. Each maps shows the locations for a single individual (seal name given in bottom right corner). Each individual’s capture site is marked with a star and locations triangulated by satellite are color-coded based on the month. Insets are provided to show the general location of the sites.
(TIFF)

Figure S4 Movement of satellite-tracked ringed seals. Each maps shows the locations for a single individual (seal name given in bottom right corner). Each individual’s capture site is marked with a star and locations triangulated by satellite are color-coded based on the month. Insets are provided to show the general location of the sites.
(TIFF)

Figure S5 Movement of satellite-tracked ringed seals. Each maps shows the locations for a single individual (seal name given in bottom right corner). Each individual’s capture site is marked with a star and locations triangulated by satellite are color-coded based on the month. Insets are provided to show the general location of the sites.
(TIFF)

Figure S6 Movement of satellite-tracked ringed seals. Each maps shows the locations for a single individual (seal name given in bottom right corner). Each individual’s capture site is marked with a star and locations triangulated by satellite are color-coded based on the month. Insets are provided to show the general location of the sites.
(TIFF)

Figure S7 Movement of satellite-tracked ringed seals. Each maps shows the locations for a single individual (seal name given in bottom right corner). Each individual’s capture site is marked with a star and locations triangulated by satellite are color-coded based on the month. Insets are provided to show the general location of the sites.
(TIFF)

Figure S8 Movement of satellite-tracked ringed seals. Each maps shows the locations for a single individual (seal name given in bottom right corner). Each individual’s capture site is marked with a star and locations triangulated by satellite are color-coded based on the month. Insets are provided to show the general location of the sites.
(TIFF)

Figure S9 Movement of satellite-tracked ringed seals. Each maps shows the locations for a single individual (seal name given in bottom right corner). Each individual’s capture site is marked with a star and locations triangulated by satellite are color-coded based on the month. Insets are provided to show the general location of the sites.
(TIFF)

Figure S10 Movement of satellite-tracked ringed seals. Each maps shows the locations for a single individual (seal name given in bottom right corner). Each individual’s capture site is marked with a star and locations triangulated by satellite are color-coded based on the month. Insets are provided to show the general location of the sites.
(TIFF)

Figure S11 Movement of satellite-tracked ringed seals. Each maps shows the locations for a single individual (seal name given in bottom right corner). Each individual’s capture site is marked with a star and locations triangulated by satellite are color-coded based on the month. Insets are provided to show the general location of the sites.
(TIFF)

Figure S12 Movement of satellite-tracked ringed seals. Each maps shows the locations for a single individual (seal name given in bottom right corner). Each individual’s capture site is marked with a star and locations triangulated by satellite are color-coded based on the month. Insets are provided to show the general location of the sites.
(TIFF)

Figure S13 Movement of satellite-tracked ringed seals. Each maps shows the locations for a single individual (seal name given in bottom right corner). Each individual’s capture site is marked with a star and locations triangulated by satellite are color-coded based on the month. Insets are provided to show the general location of the sites.
(TIFF)

Figure S14 Movement of satellite-tracked ringed seals. Each maps shows the locations for a single individual (seal name given in bottom right corner). Each individual’s capture site is marked with a star and locations triangulated by satellite are color-coded based on the month. Insets are provided to show the general location of the sites.
(TIFF)

Figure S15 Movement of satellite-tracked ringed seals. Each maps shows the locations for a single individual (seal name given in bottom right corner). Each individual’s capture site is marked with a star and locations triangulated by satellite are color-coded based on the month. Insets are provided to show the general location of the sites.
(TIFF)

Figure S16 Movement of satellite-tracked ringed seals. Each maps shows the locations for a single individual (seal name given in bottom right corner). Each individual’s capture site is marked with a star and locations triangulated by satellite are color-coded based on the month. Insets are provided to show the general location of the sites.
(TIFF)

Figure S17 Maximum Likelihood phylogeny based on the mtDNA Control Region. Individuals are color-coded based on their breeding site. Only bootstrap values > 50% are shown. Each individual had a unique CR haplotype, and there was clear clustering of individuals from Lake Saimaa but minimal or no phylogeographic clustering for the Baltic or Arctic breeding sites. Note, Ulukhaktok/Holman is denoted as Holman.
(TIF)

Figure S18 Maximum Likelihood phylogeny based on the mtDNA Cytochrome Oxidase I. There were 31 unique COI haplotypes among the 113 individuals sequenced; all individuals were included in the phylogeny. The individuals in Lake Saimaa clustered by haplotype, but there was vey little clustering of individuals from other breeding sites. Bootstrap values > 50% are shown and individuals are color-coded by breeding site. Note, Ulukhaktok/Holman is denoted as Holman.
(TIF)

Figures S19 Expected and Observed heterozygosity for each locus and breeding site. Each plot shows the expected (triangles) and observed (circles) heterozygosity for a single microsatellite locus at each sample site. Sample sites are arranged from left to right on the x-axis based on their geographic position, west to east. The coloring indicates the p-value of the test for HWE. Note, Ulukhaktok/Holman is denoted as Holman.
(TIFF)

Figure S20 Expected and Observed heterozygosity for each locus and breeding site. Each plot shows the expected (triangles) and observed (circles) heterozygosity for a single microsatellite locus at each sample site. Sample sites are arranged from left to right on the x-axis based on their geographic position, west to east. The coloring indicates the p-value of the test for HWE. Note, Ulukhaktok/Holman is denoted as Holman.
(TIFF)

Figure S21 **Normalized heterozygosity by sample site excluding SGPV16.** The mean normalized heterozygosity +/– SD for all loci with the exclusion of SGPV16. The expected and observed heterozygosity for each locus was normalized by the maximum. Triangles are expected heterozygosity and circles are observed. Sample sites are arranged from left to right on the x-axis based on their geographic position, west to east. The Arctic subspecies is colored purple, the Baltic subspecies is maroon, and the Lake Saimaa subspecies is black. Note, Ulukhaktok/Holman is denoted as Holman.
(TIFF)

Figure S22 Normalized heterozygosity by sample site with SGPV16 and other loci excluded. The mean normalized heterozygosity +/– SD for all loci with the exclusion of SGPV16 and an additional locus. The expected and observed heterozygosity for each locus was normalized by the maximum. Triangles are expected heterozygosity and circles are observed. Sample sites are arranged from left to right on the x-axis based on their geographic position, west to east. The Arctic subspecies is colored purple, the Baltic subspecies is maroon, and the Lake Saimaa subspecies is black. Note, Ulukhaktok/Holman is denoted as Holman.
(TIFF)

Figure S23 Normalized heterozygosity by sample site with SGPV16 and other loci excluded. The mean normalized heterozygosity +/– SD for all loci with the exclusion of SGPV16 and an additional locus. The expected and observed heterozygosity for each locus was normalized by the maximum. Triangles are expected heterozygosity and circles are observed. Sample sites are arranged from left to right on the x-axis based on their geographic position, west to east. The Arctic subspecies is colored purple, the Baltic subspecies is maroon, and the Lake Saimaa subspecies is black. Note, Ulukhaktok/Holman is denoted as Holman.
(TIFF)

Figure S24 Normalized heterozygosity by sample site with SGPV16 and other loci excluded. The mean normalized heterozygosity +/– SD for all loci with the exclusion of SGPV16 and an additional locus. The expected and observed heterozygosity for each locus was normalized by the maximum. Triangles are expected heterozygosity and circles are observed. Sample sites are arranged from left to right on the x-axis based on their geographic position, west to east. The Arctic subspecies is colored purple, the Baltic subspecies is maroon, and the Lake Saimaa subspecies is black. Note, Ulukhaktok/Holman is denoted as Holman.
(TIFF)

Figure S25 Normalized heterozygosity by sample site with SGPV16 and other loci excluded. The mean normalized heterozygosity +/– SD for all loci with the exclusion of SGPV16 and an additional locus. The expected and observed heterozygosity for each locus was normalized by the maximum. Triangles are expected heterozygosity and circles are observed. Sample sites are arranged from left to right on the x-axis based on their geographic position, west to east. The Arctic subspecies is colored purple, the Baltic subspecies is maroon, and the Lake Saimaa subspecies is black. Note, Ulukhaktok/Holman is denoted as Holman.
(TIFF)

Figure S26 DNA extraction purity for shed-skin samples. Using a subset of the shed-skin samples collected in the Chukchi and Western Beaufort, DNA extraction quality was measured with an Eppendorf BioPhotometer. Boxplots show the distribution of the DNA purity by sample site. Pure DNA samples produce a 260/280 purity value of 1.8 (red line). A mean value of 1.6 (green) is typical for tissue samples and 1.1 (blue) for shed epidermis [25].
(TIFF)

Figure S27 Travel vs. Age. Maximum distance travelled from capture site by age and sex.
(TIFF)

File S1 Supplementary AMOVA Tables. Population pairwise F-statistics and p-values for each AMOVA.
(TXT)

File S2 Heterozygosity for each population and microsatellite locus. Expected and observed heterozygosity for each population and locus, along with p-vales from the test for HWE.
(TXT)

File S3 Migrate Profile Likelihoods. Summary of profile likelihood percentiles of all parameters for the mtDNA-based Migrate analysis and the microsatellite-based analysis.
(TXT)
