# Supplementary figures and images for: Combined Genetic and Telemetry Data Reveal High Rates of Gene Flow, Migration, and Long-Distance Dispersal Potential in Arctic Ringed Seals (Pusa hispida)
Source: PLoS One. 2013 Oct 10;8(10):e77125. doi: 10.1371/journal.pone.0077125 (PMC3794998; doi:10.1371/journal.pone.0077125)

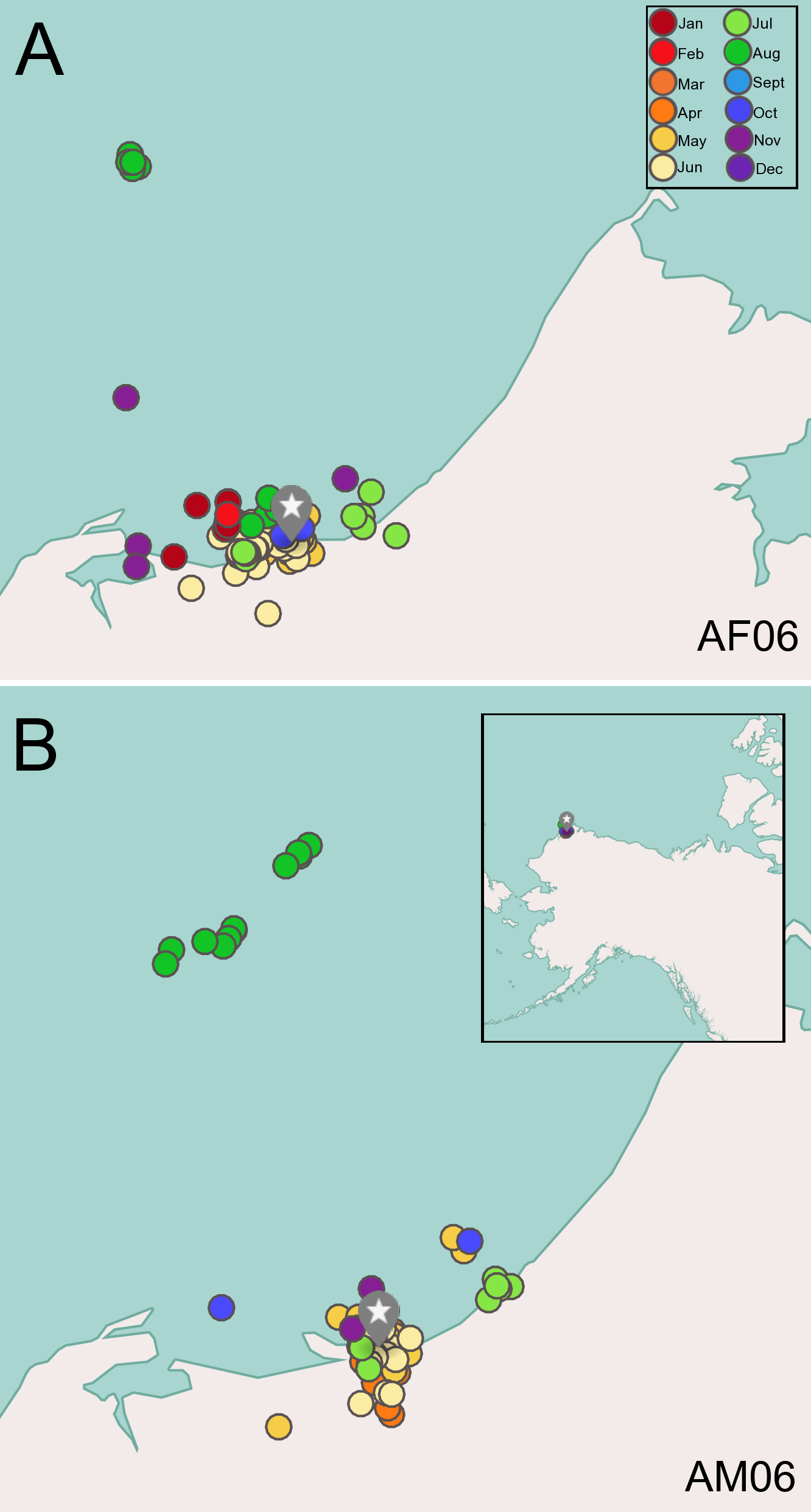

Supplement: Figure S1 — Movement of satellite-tracked ringed seals. Each maps shows the locations for a single individual (seal name given in bottom right corner). Each individual’s capture site is marked with a star and locations triangulated by satellite are color-coded based on the month. Insets are provided to show the general location of the sites. (TIFF) [file pone.0077125.s001.tif]

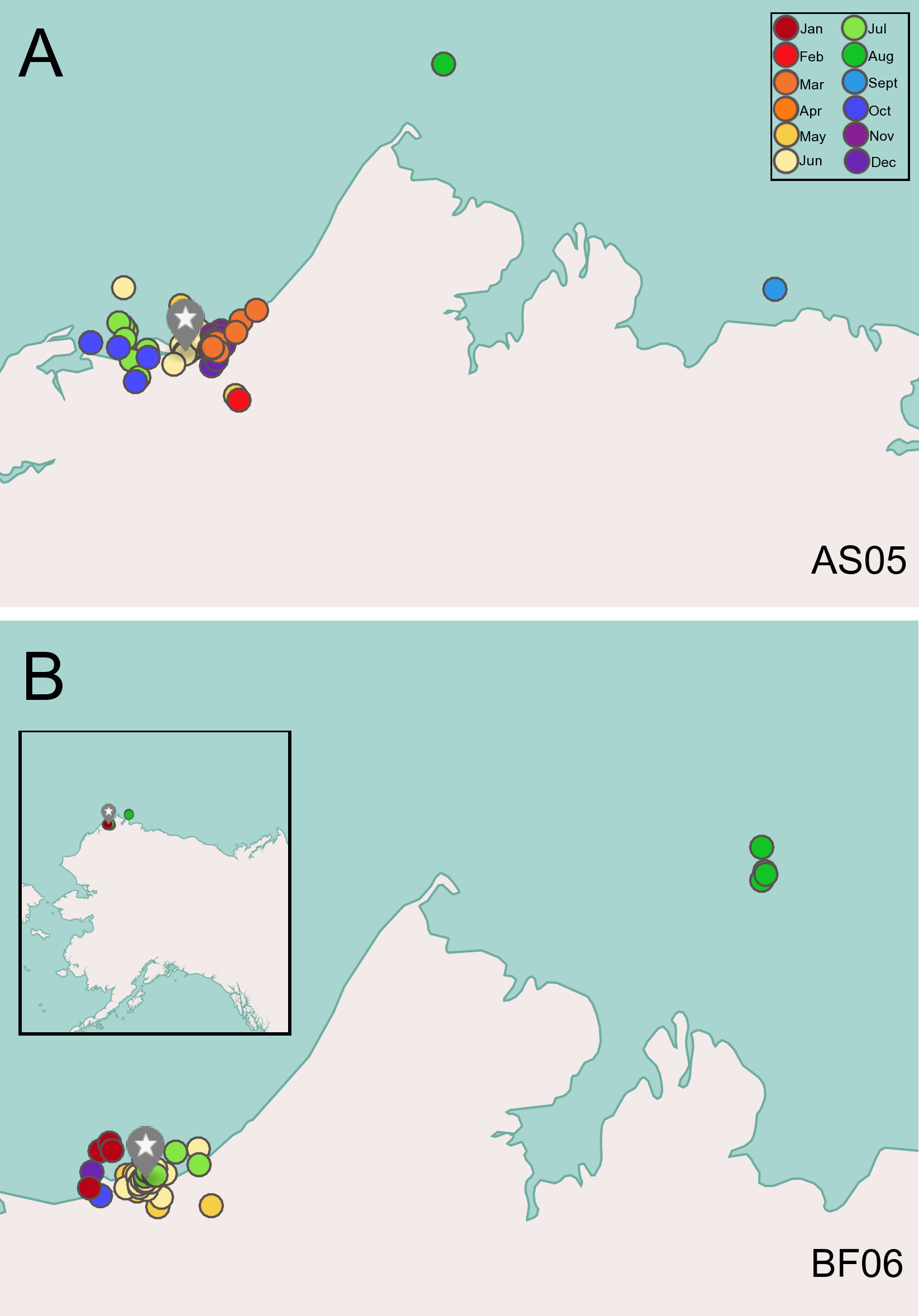

Supplement: Figure S2 — Movement of satellite-tracked ringed seals. Each maps shows the locations for a single individual (seal name given in bottom right corner). Each individual’s capture site is marked with a star and locations triangulated by satellite are color-coded based on the month. Insets are provided to show the general location of the sites. (TIFF) [file pone.0077125.s002.tif]

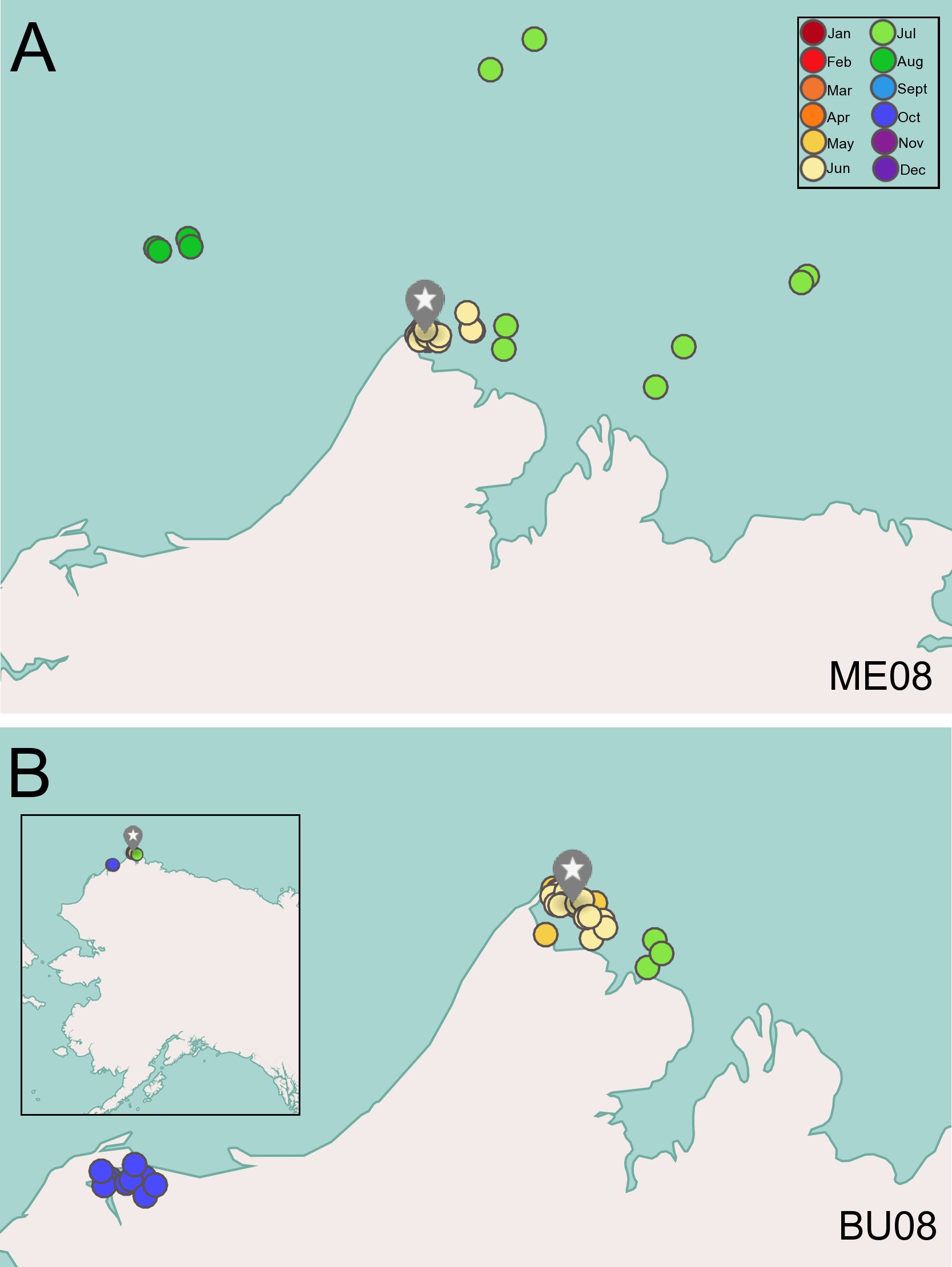

Supplement: Figure S3 — Movement of satellite-tracked ringed seals. Each maps shows the locations for a single individual (seal name given in bottom right corner). Each individual’s capture site is marked with a star and locations triangulated by satellite are color-coded based on the month. Insets are provided to show the general location of the sites. (TIFF) [file pone.0077125.s003.tif]

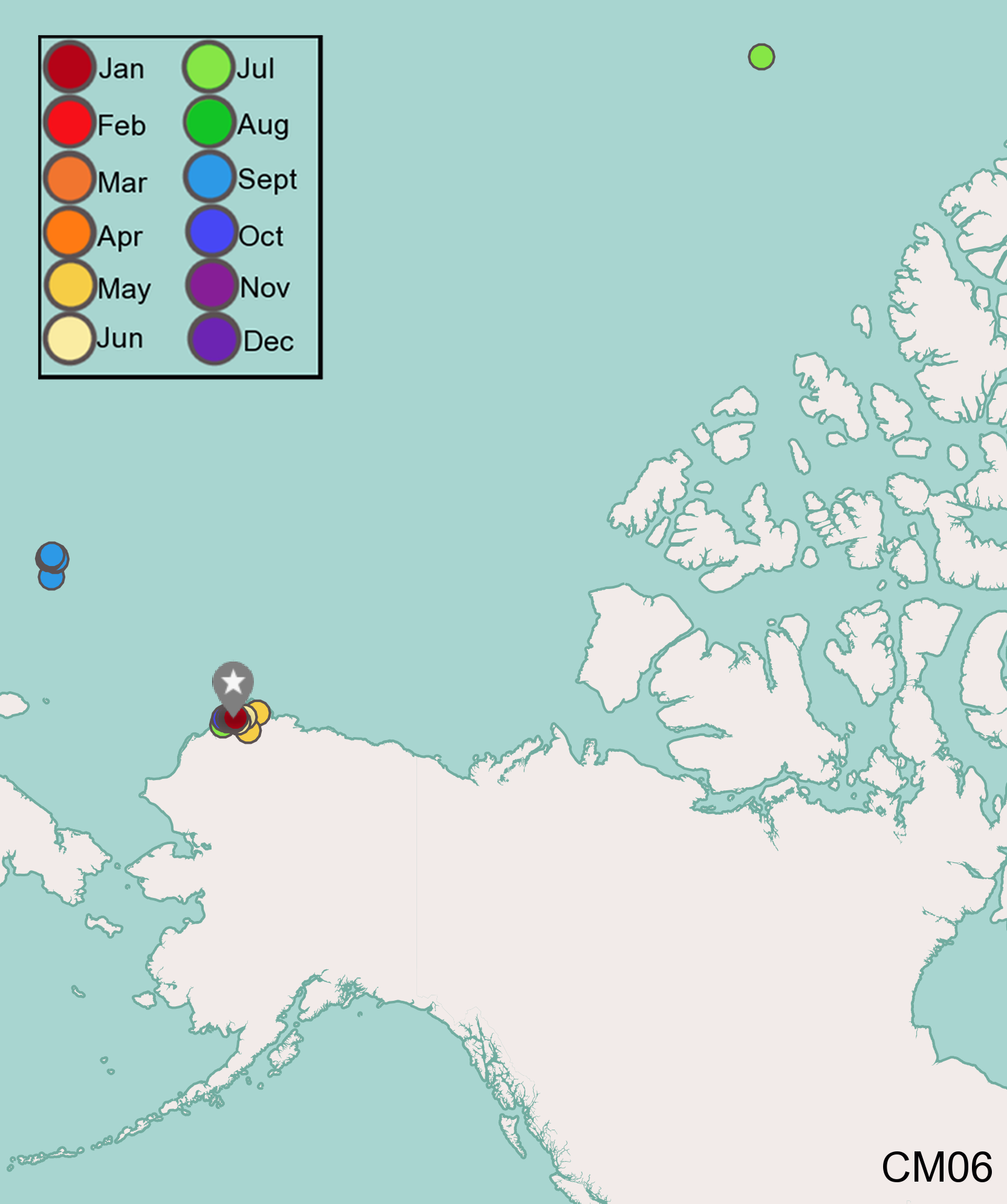

Supplement: Figure S4 — Movement of satellite-tracked ringed seals. Each maps shows the locations for a single individual (seal name given in bottom right corner). Each individual’s capture site is marked with a star and locations triangulated by satellite are color-coded based on the month. Insets are provided to show the general location of the sites. (TIFF) [file pone.0077125.s004.tif]

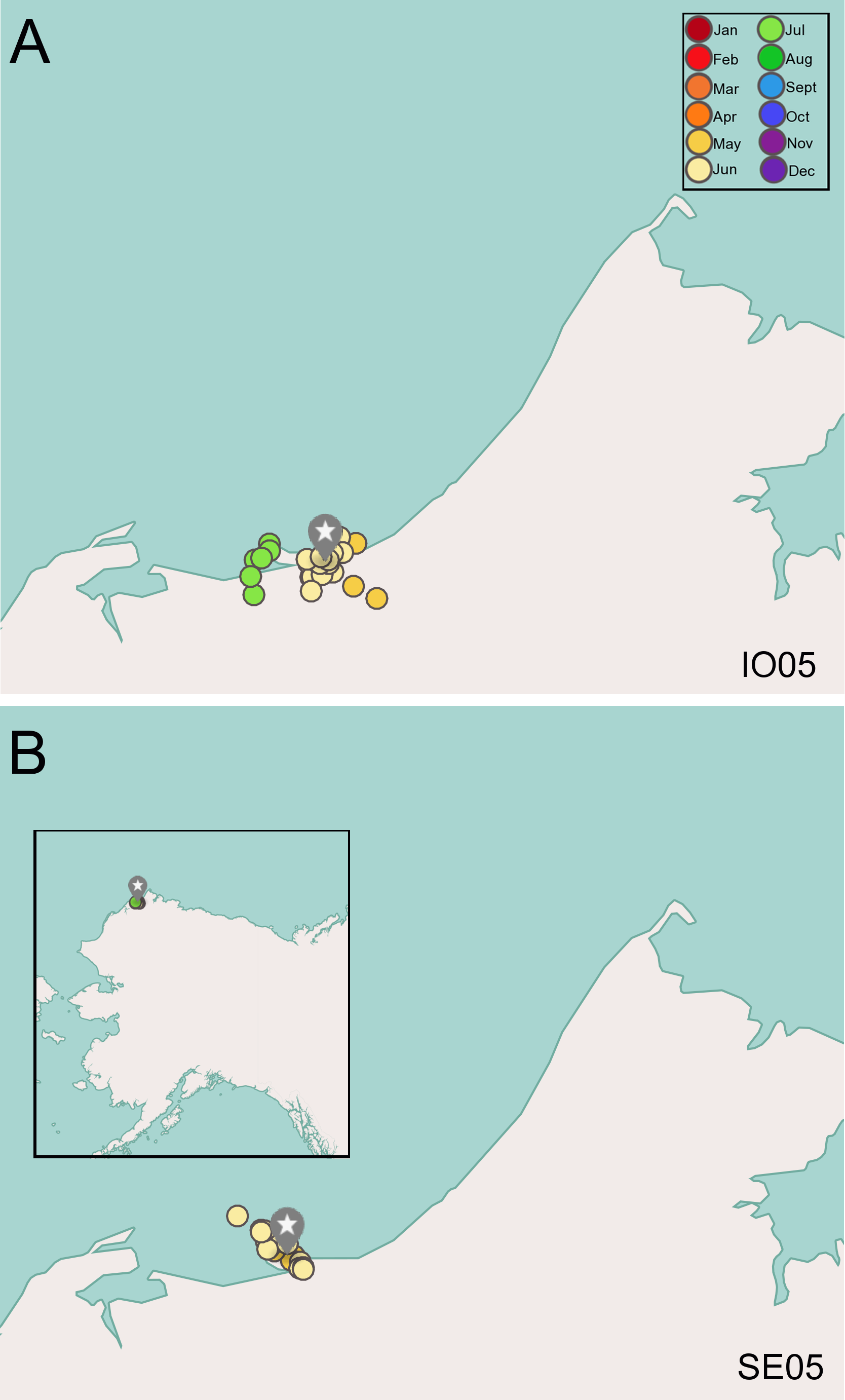

Supplement: Figure S5 — Movement of satellite-tracked ringed seals. Each maps shows the locations for a single individual (seal name given in bottom right corner). Each individual’s capture site is marked with a star and locations triangulated by satellite are color-coded based on the month. Insets are provided to show the general location of the sites. (TIFF) [file pone.0077125.s005.tif]

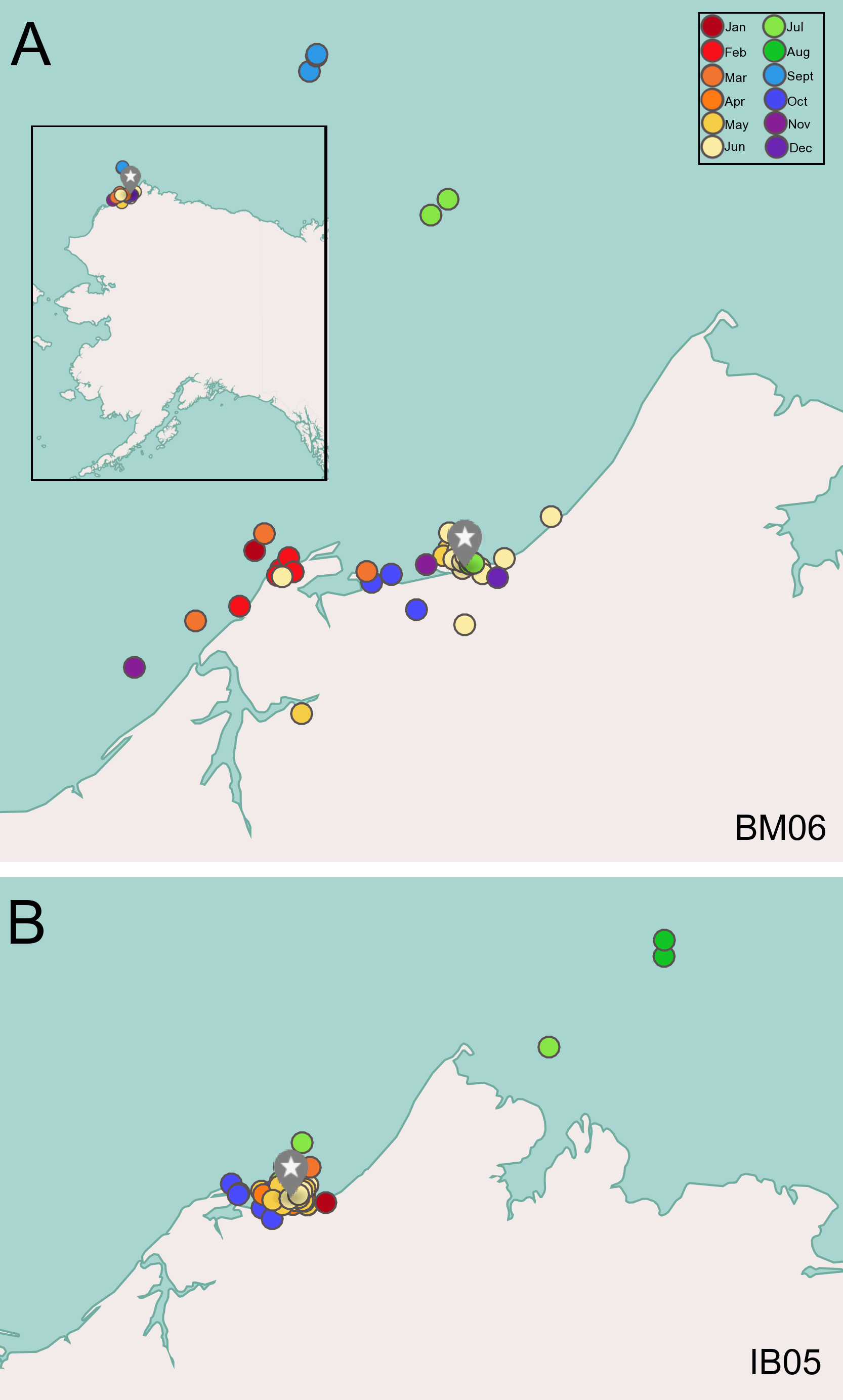

Supplement: Figure S6 — Movement of satellite-tracked ringed seals. Each maps shows the locations for a single individual (seal name given in bottom right corner). Each individual’s capture site is marked with a star and locations triangulated by satellite are color-coded based on the month. Insets are provided to show the general location of the sites. (TIFF) [file pone.0077125.s006.tif]

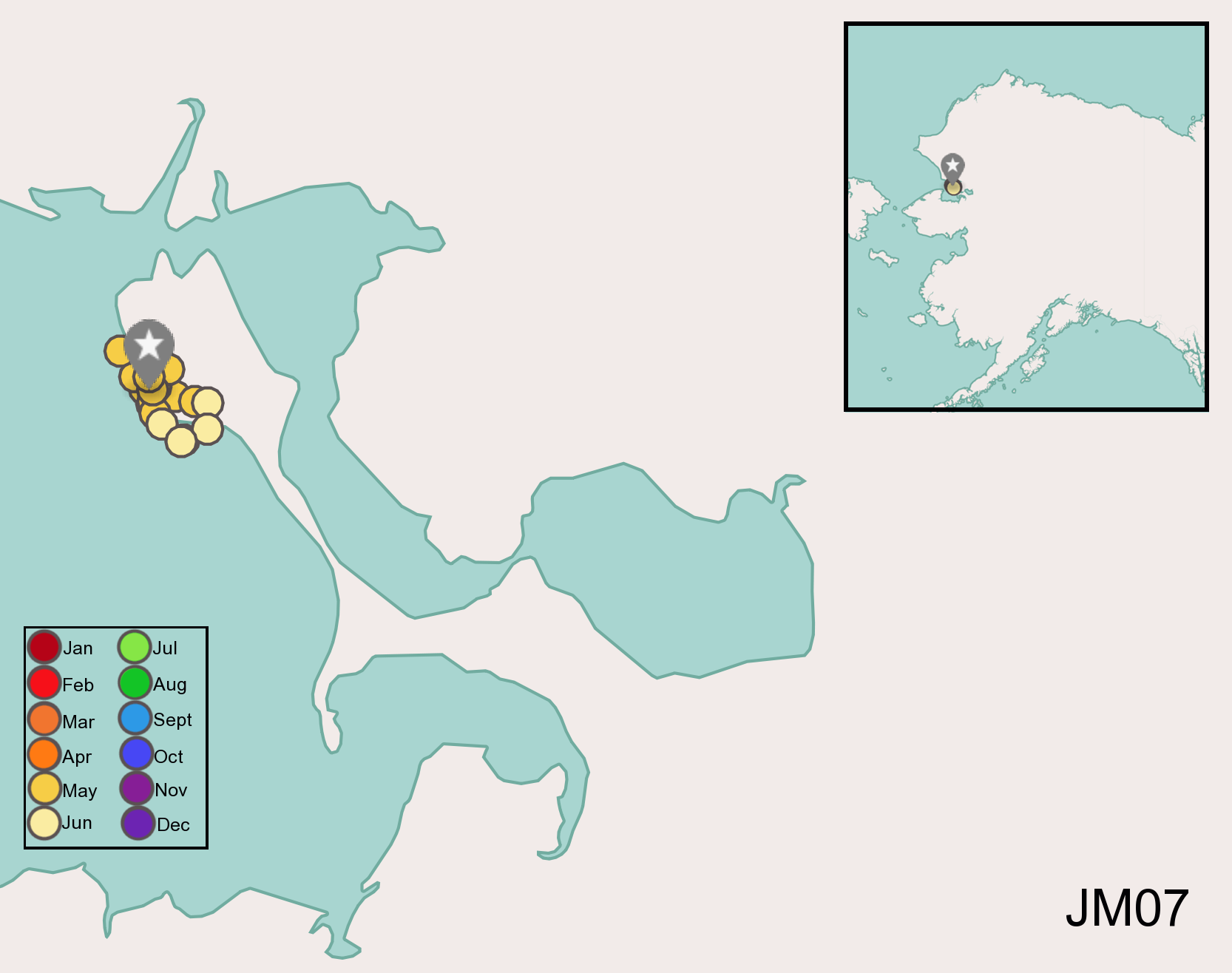

Supplement: Figure S7 — Movement of satellite-tracked ringed seals. Each maps shows the locations for a single individual (seal name given in bottom right corner). Each individual’s capture site is marked with a star and locations triangulated by satellite are color-coded based on the month. Insets are provided to show the general location of the sites. (TIFF) [file pone.0077125.s007.tif]

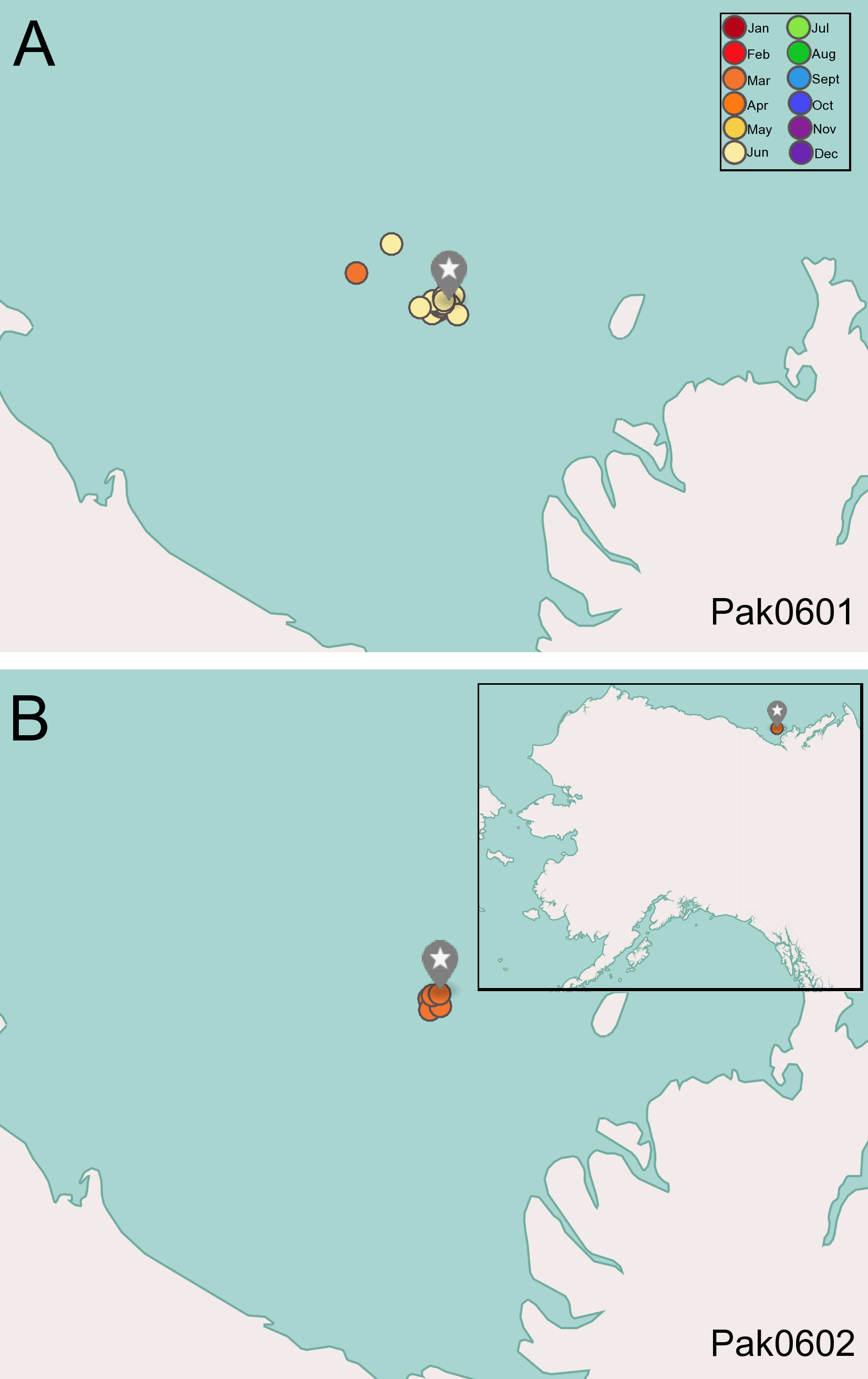

Supplement: Figure S8 — Movement of satellite-tracked ringed seals. Each maps shows the locations for a single individual (seal name given in bottom right corner). Each individual’s capture site is marked with a star and locations triangulated by satellite are color-coded based on the month. Insets are provided to show the general location of the sites. (TIFF) [file pone.0077125.s008.tif]

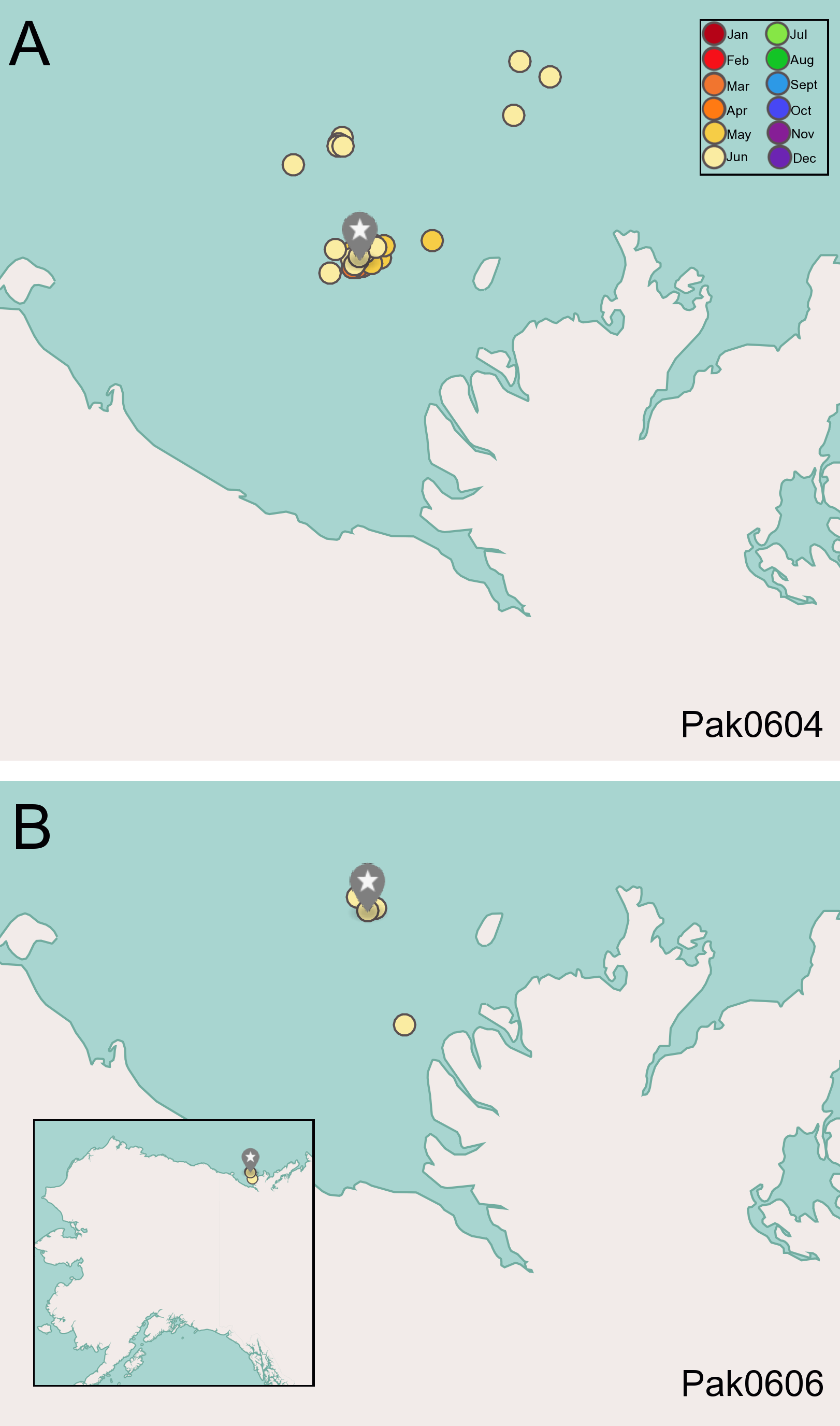

Supplement: Figure S9 — Movement of satellite-tracked ringed seals. Each maps shows the locations for a single individual (seal name given in bottom right corner). Each individual’s capture site is marked with a star and locations triangulated by satellite are color-coded based on the month. Insets are provided to show the general location of the sites. (TIFF) [file pone.0077125.s009.tif]

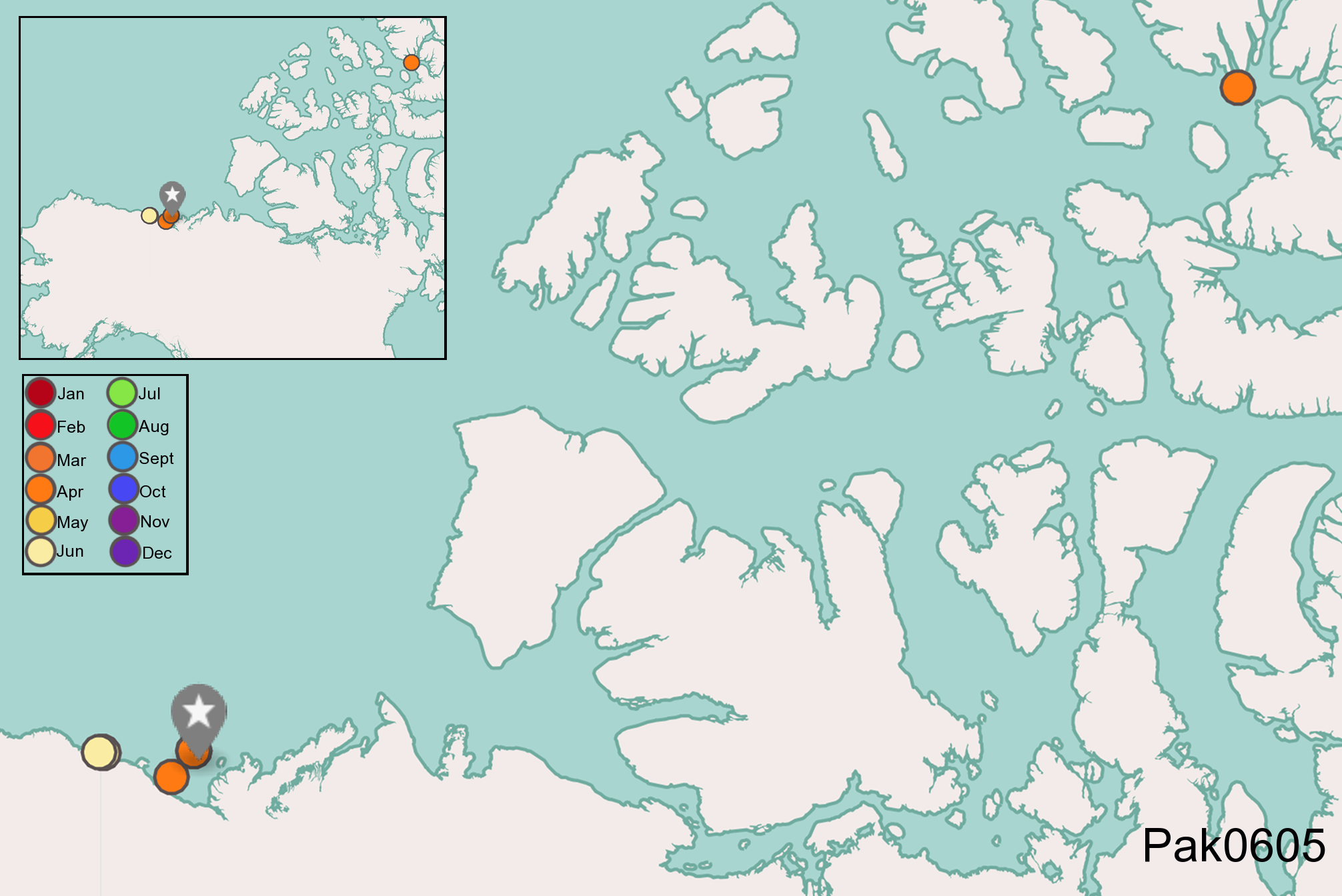

Supplement: Figure S10 — Movement of satellite-tracked ringed seals. Each maps shows the locations for a single individual (seal name given in bottom right corner). Each individual’s capture site is marked with a star and locations triangulated by satellite are color-coded based on the month. Insets are provided to show the general location of the sites. (TIFF) [file pone.0077125.s010.tif]

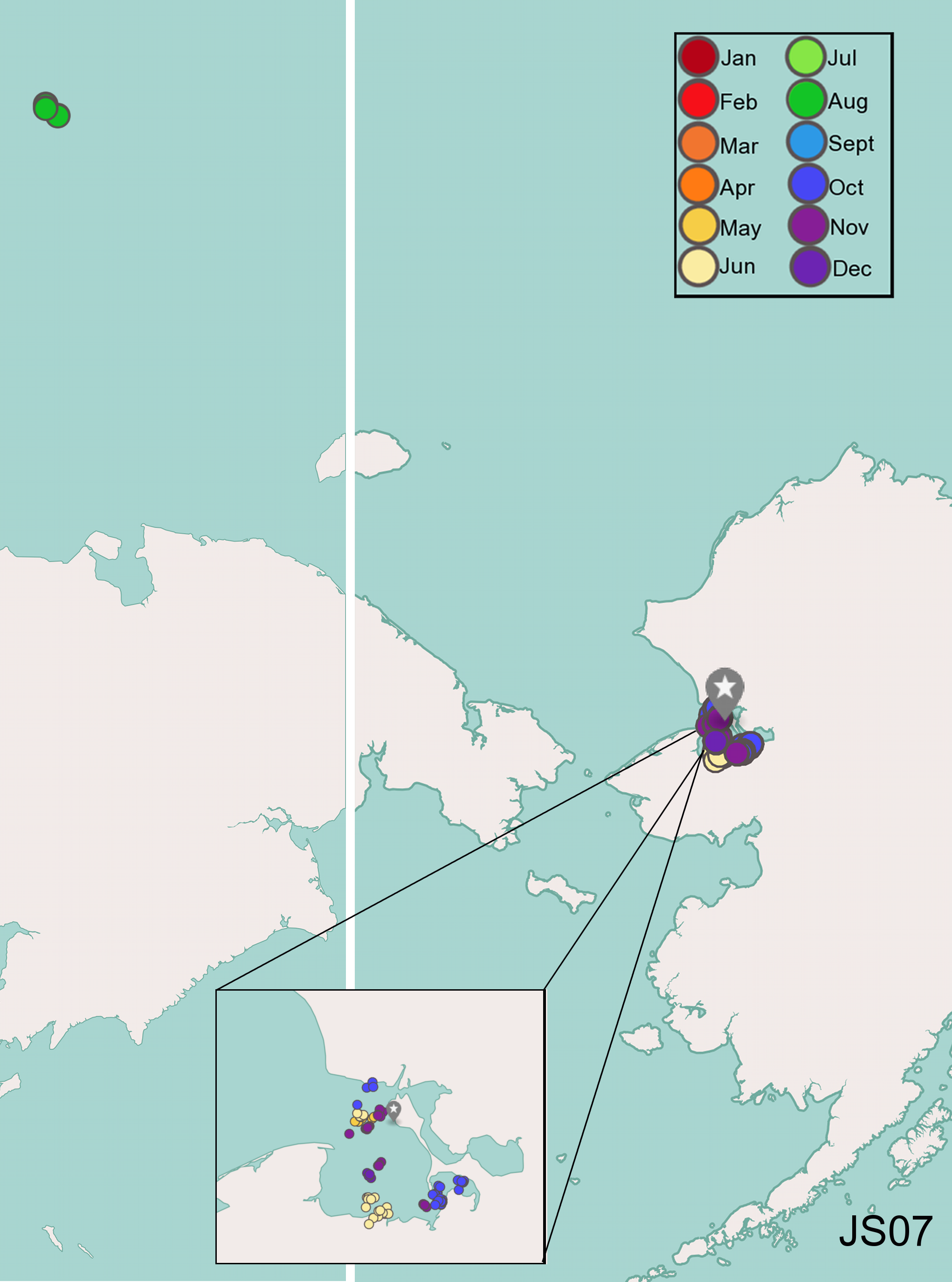

Supplement: Figure S11 — Movement of satellite-tracked ringed seals. Each maps shows the locations for a single individual (seal name given in bottom right corner). Each individual’s capture site is marked with a star and locations triangulated by satellite are color-coded based on the month. Insets are provided to show the general location of the sites. (TIFF) [file pone.0077125.s011.tif]

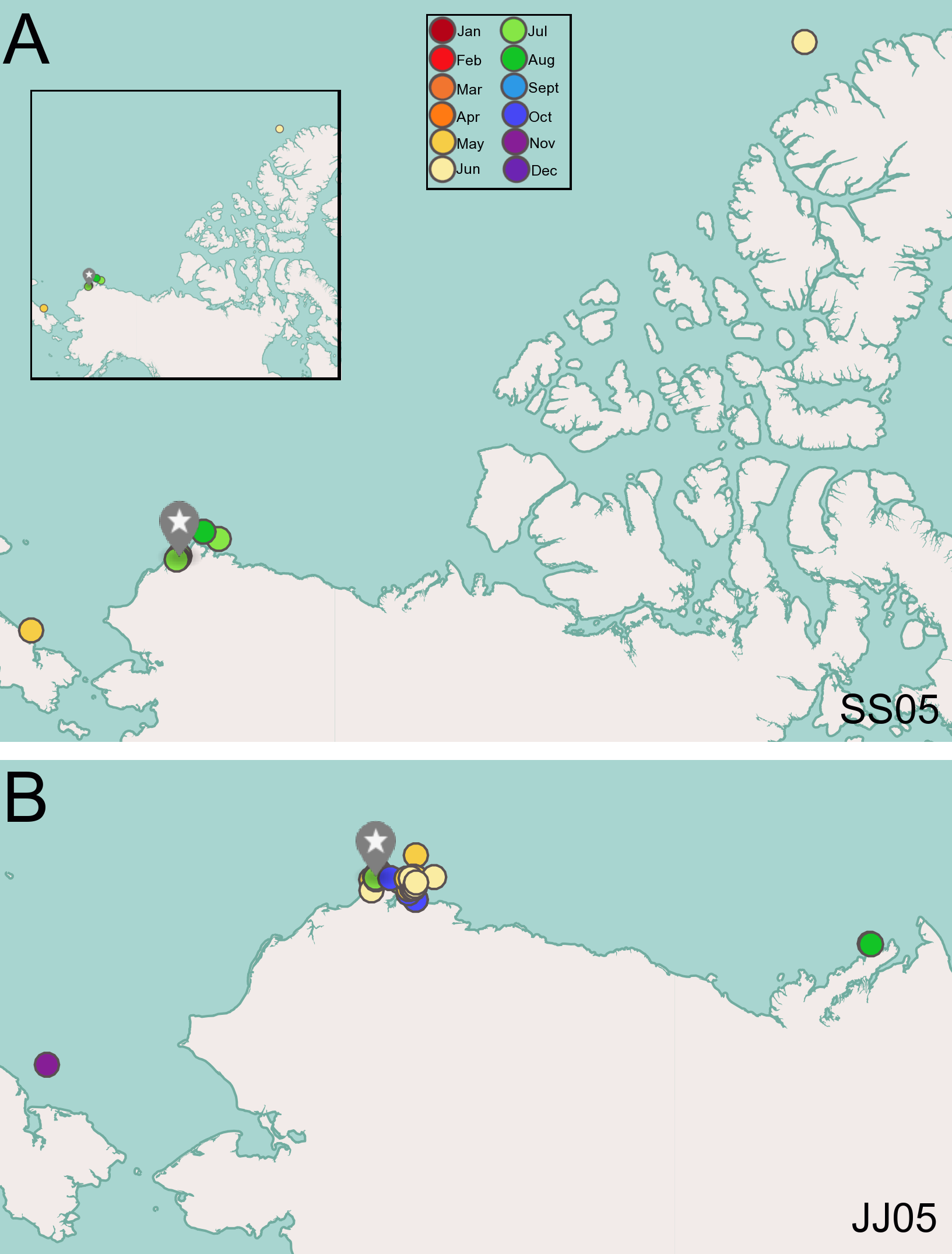

Supplement: Figure S12 — Movement of satellite-tracked ringed seals. Each maps shows the locations for a single individual (seal name given in bottom right corner). Each individual’s capture site is marked with a star and locations triangulated by satellite are color-coded based on the month. Insets are provided to show the general location of the sites. (TIFF) [file pone.0077125.s012.tif]

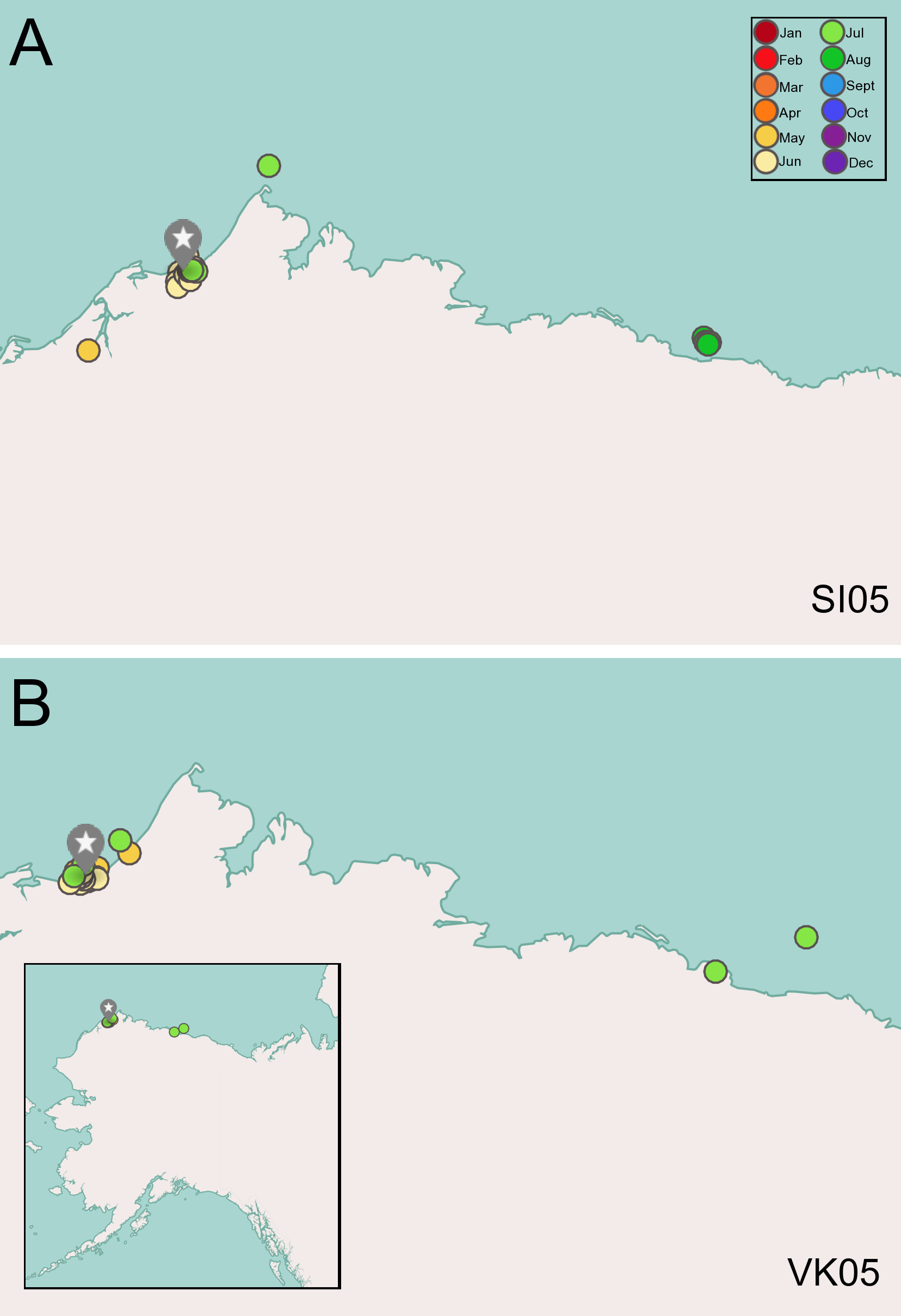

Supplement: Figure S13 — Movement of satellite-tracked ringed seals. Each maps shows the locations for a single individual (seal name given in bottom right corner). Each individual’s capture site is marked with a star and locations triangulated by satellite are color-coded based on the month. Insets are provided to show the general location of the sites. (TIFF) [file pone.0077125.s013.tif]

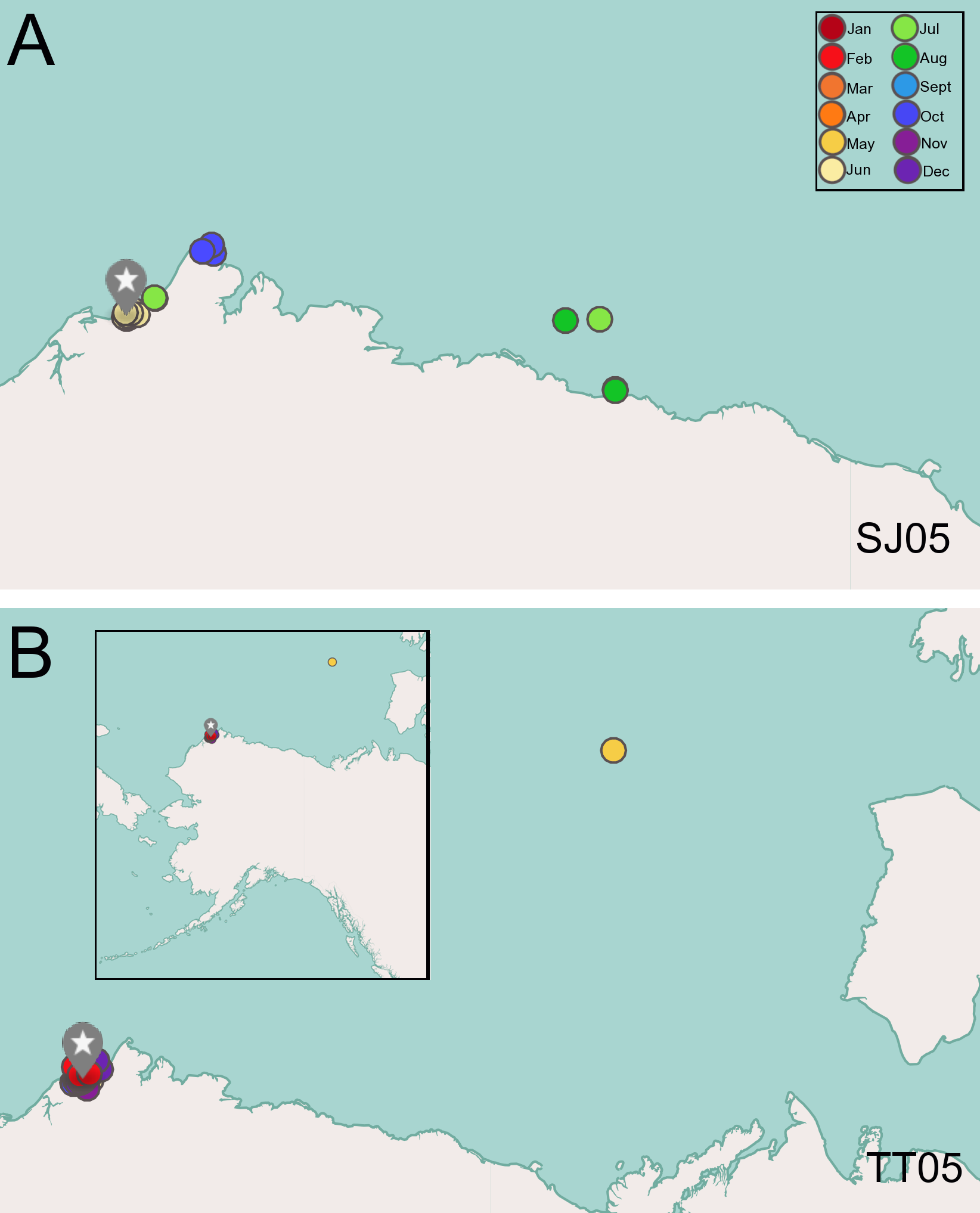

Supplement: Figure S14 — Movement of satellite-tracked ringed seals. Each maps shows the locations for a single individual (seal name given in bottom right corner). Each individual’s capture site is marked with a star and locations triangulated by satellite are color-coded based on the month. Insets are provided to show the general location of the sites. (TIFF) [file pone.0077125.s014.tif]

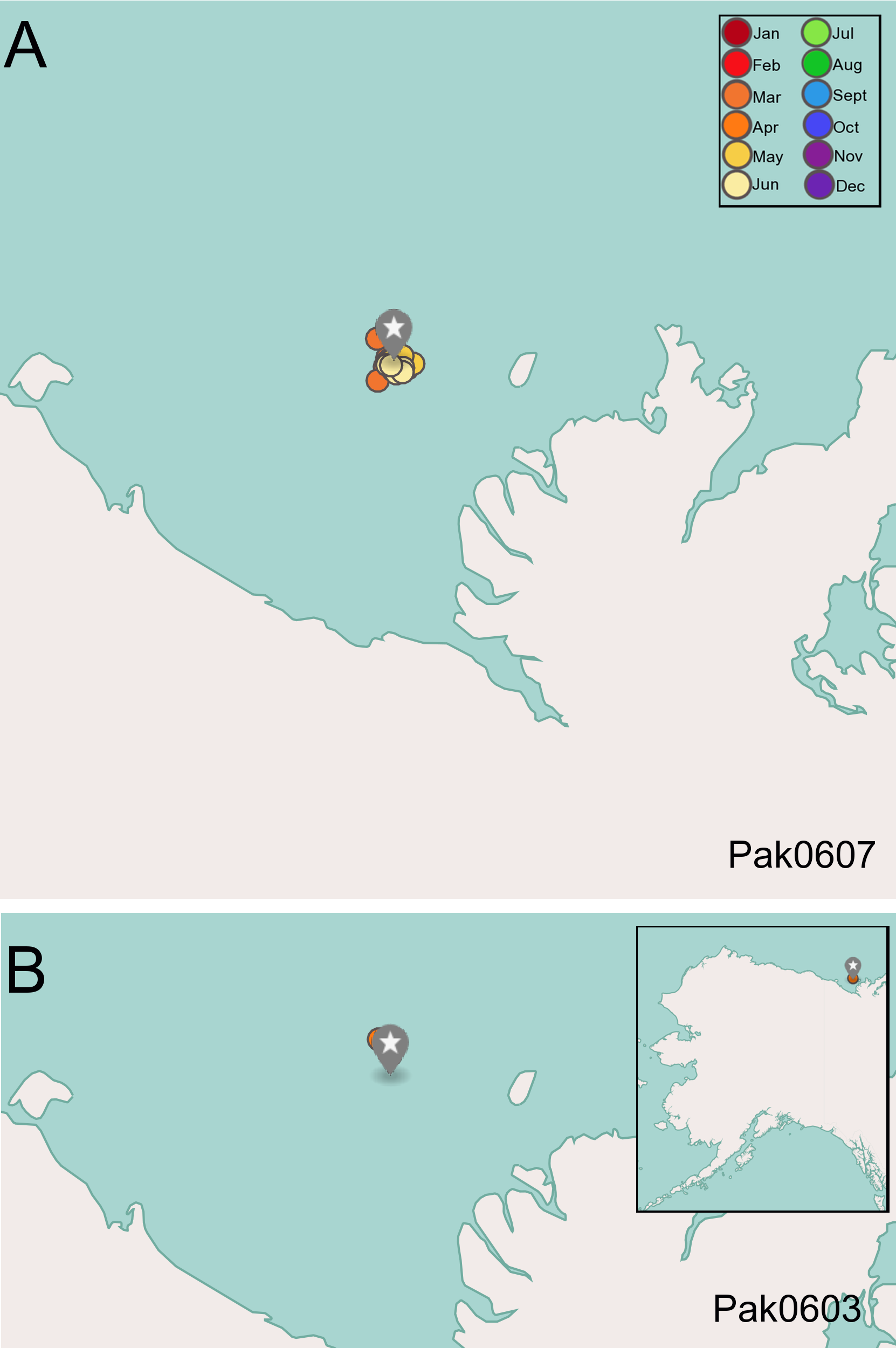

Supplement: Figure S15 — Movement of satellite-tracked ringed seals. Each maps shows the locations for a single individual (seal name given in bottom right corner). Each individual’s capture site is marked with a star and locations triangulated by satellite are color-coded based on the month. Insets are provided to show the general location of the sites. (TIFF) [file pone.0077125.s015.tif]

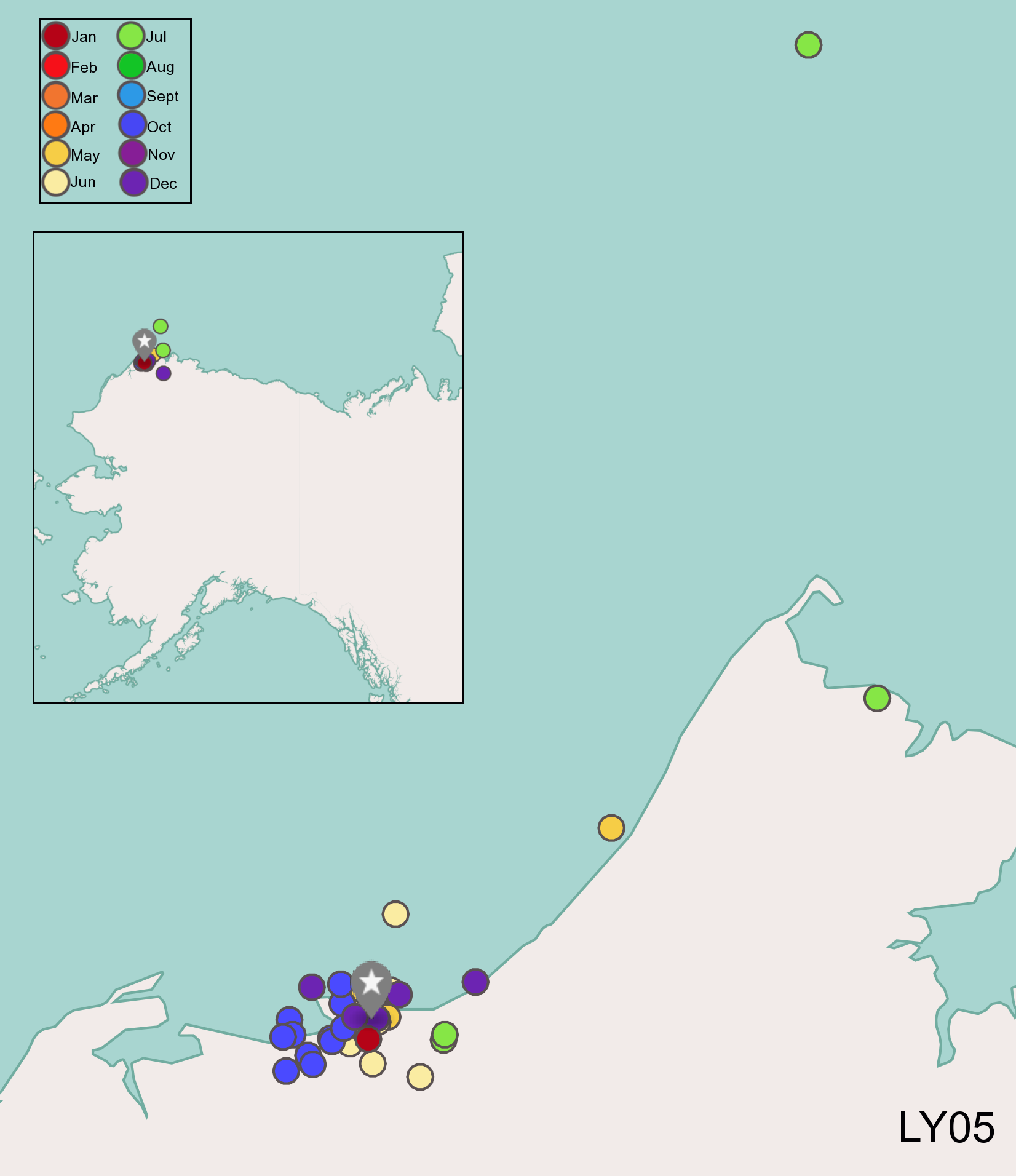

Supplement: Figure S16 — Movement of satellite-tracked ringed seals. Each maps shows the locations for a single individual (seal name given in bottom right corner). Each individual’s capture site is marked with a star and locations triangulated by satellite are color-coded based on the month. Insets are provided to show the general location of the sites. (TIFF) [file pone.0077125.s016.tif]

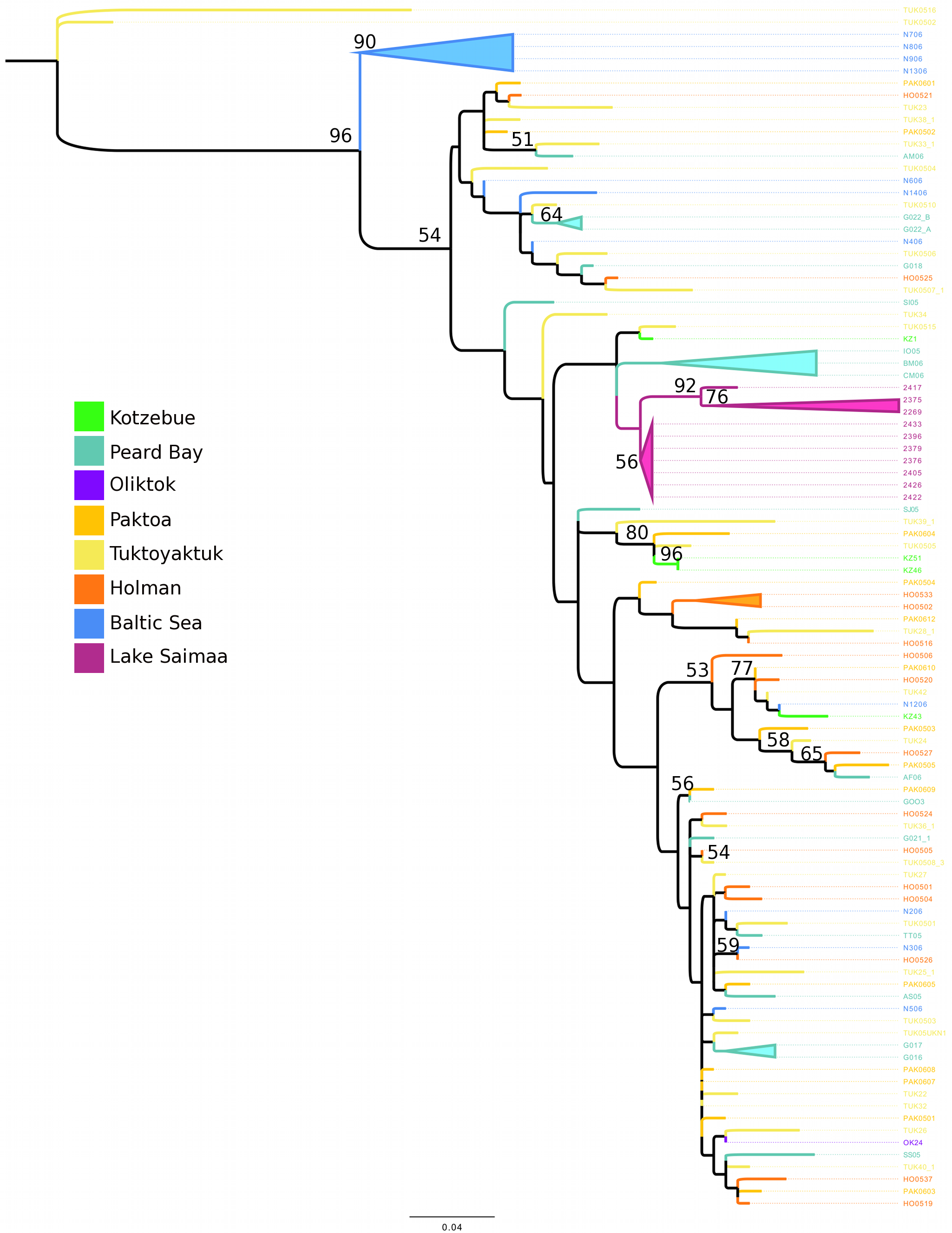

Supplement: Figure S17 — Maximum Likelihood phylogeny based on the mtDNA Control Region. Individuals are color-coded based on their breeding site. Only bootstrap values > 50% are shown. Each individual had a unique CR haplotype, and there was clear clustering of individuals from Lake Saimaa but minimal or no phylogeographic clustering for the Baltic or Arctic breeding sites. Note, Ulukhaktok/Holman is denoted as Holman. (TIF) [file pone.0077125.s017.tif]

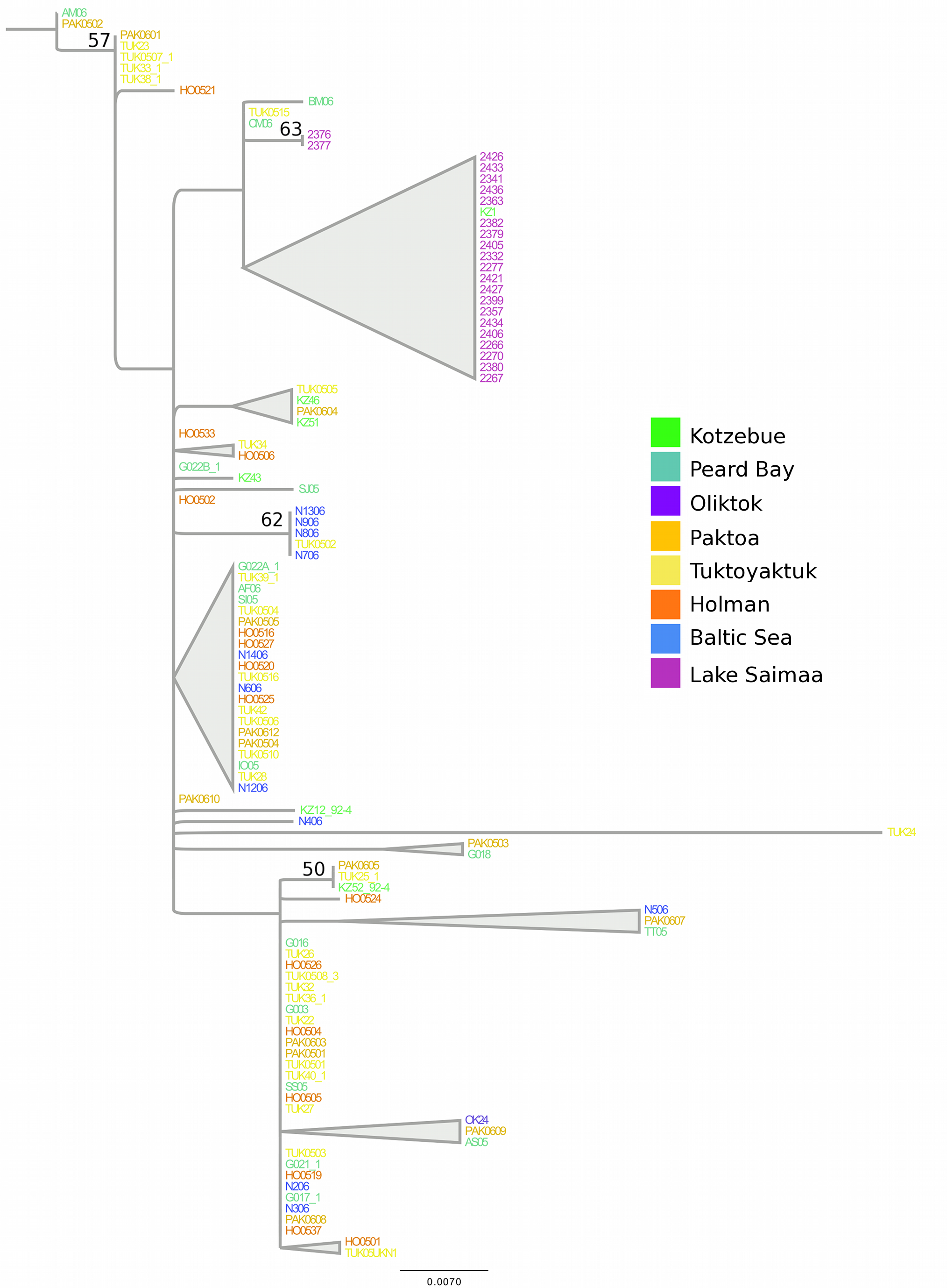

Supplement: Figure S18 — Maximum Likelihood phylogeny based on the mtDNA Cytochrome Oxidase I. There were 31 unique COI haplotypes among the 113 individuals sequenced; all individuals were included in the phylogeny. The individuals in Lake Saimaa clustered by haplotype, but there was vey little clustering of individuals from other breeding sites. Bootstrap values > 50% are shown and individuals are color-coded by breeding site. Note, Ulukhaktok/Holman is denoted as Holman. (TIF) [file pone.0077125.s018.tif]

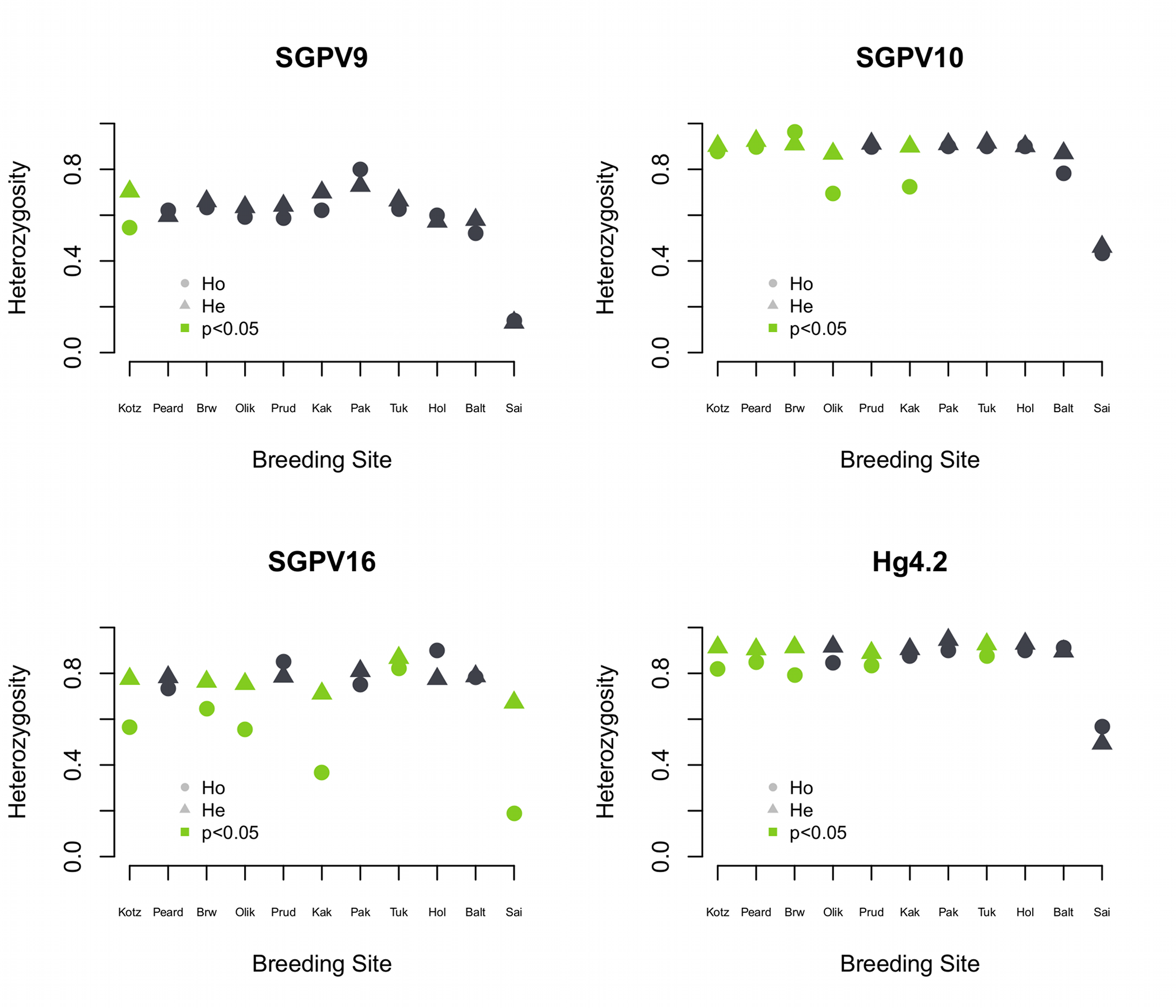

Supplement: Figures S19 — Expected and Observed heterozygosity for each locus and breeding site. Each plot shows the expected (triangles) and observed (circles) heterozygosity for a single microsatellite locus at each sample site. Sample sites are arranged from left to right on the x-axis based on their geographic position, west to east. The coloring indicates the p-value of the test for HWE. Note, Ulukhaktok/Holman is denoted as Holman. (TIFF) [file pone.0077125.s019.tif]

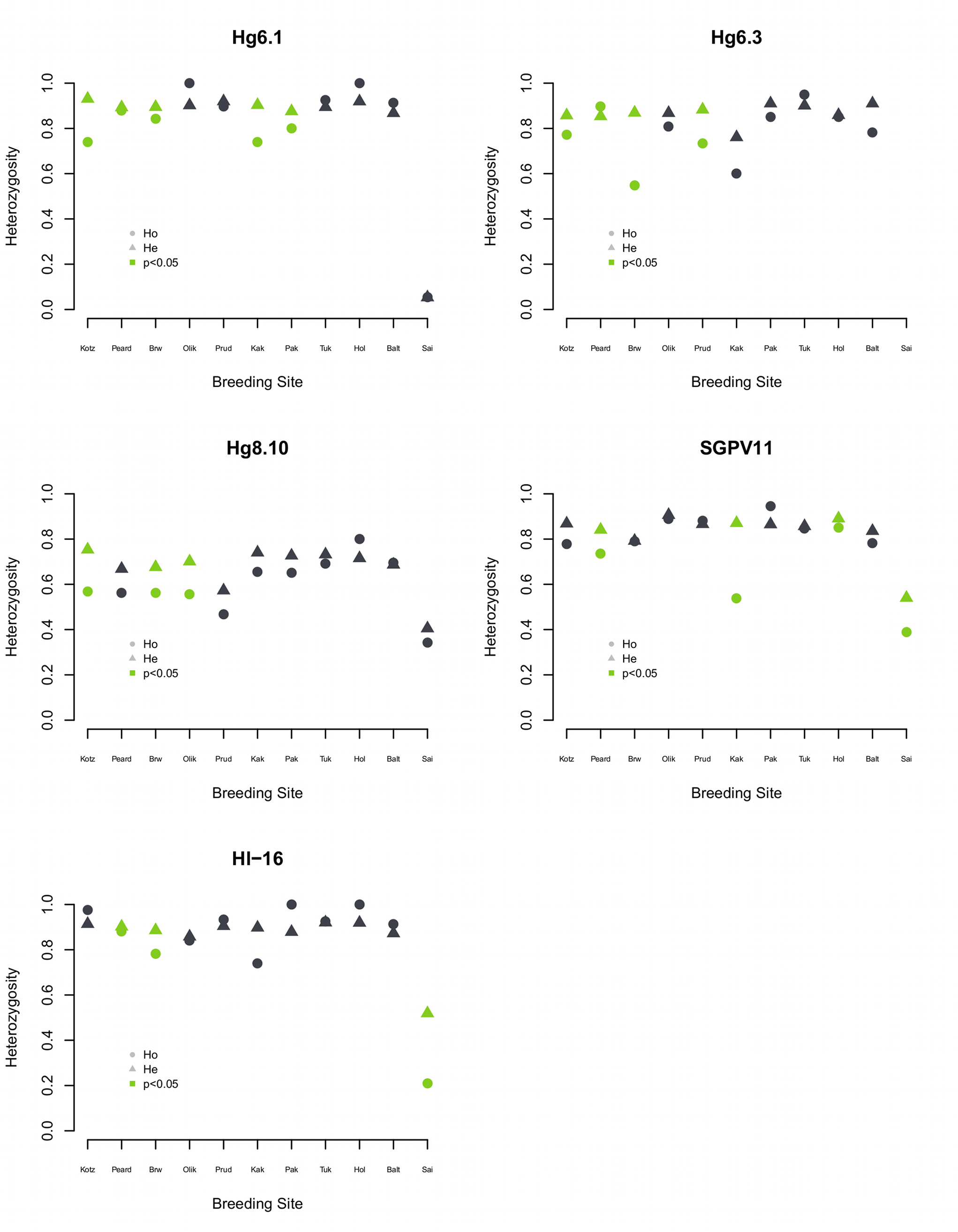

Supplement: Figure S20 — Expected and Observed heterozygosity for each locus and breeding site. Each plot shows the expected (triangles) and observed (circles) heterozygosity for a single microsatellite locus at each sample site. Sample sites are arranged from left to right on the x-axis based on their geographic position, west to east. The coloring indicates the p-value of the test for HWE. Note, Ulukhaktok/Holman is denoted as Holman. (TIFF) [file pone.0077125.s020.tif]

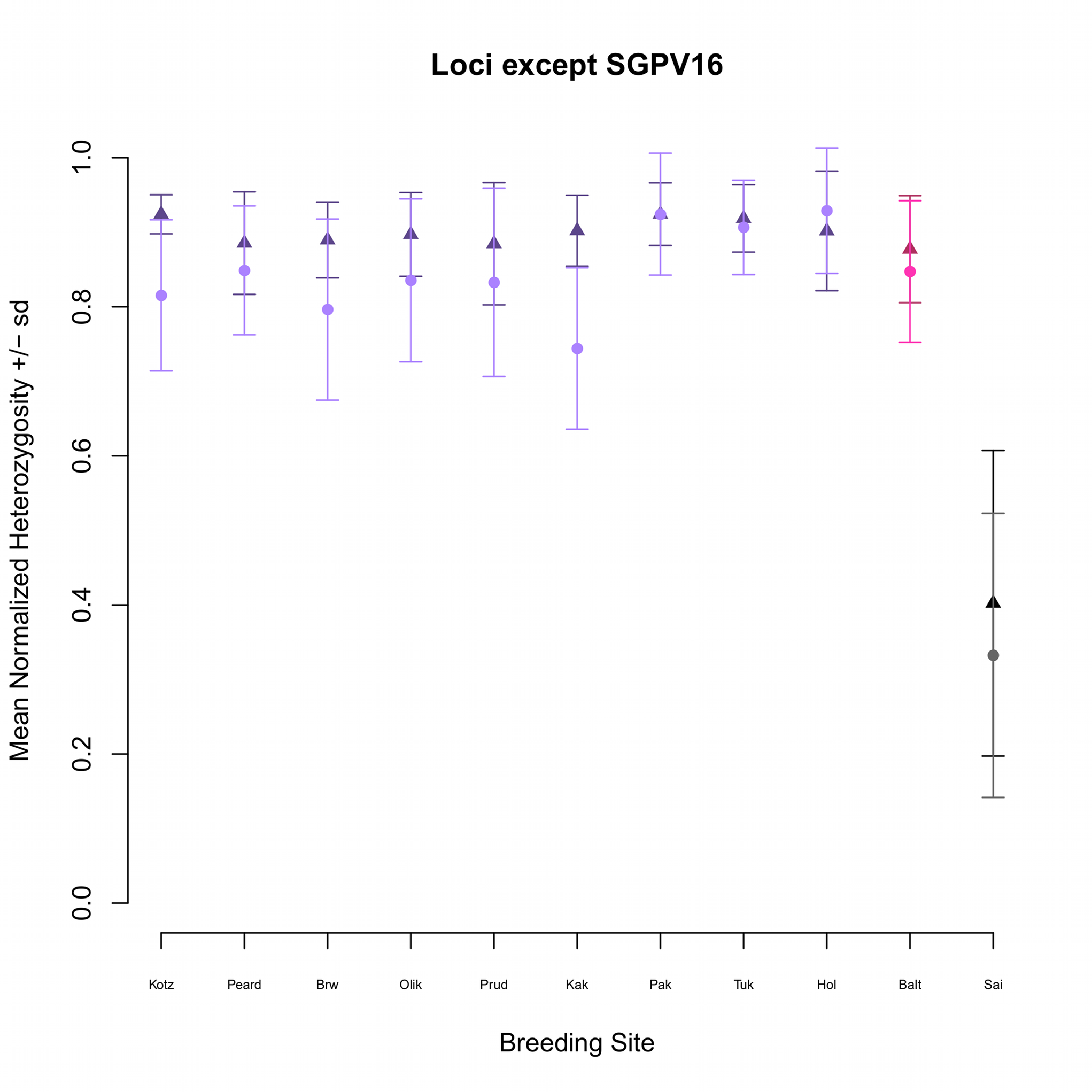

Supplement: Figure S21 — Normalized heterozygosity by sample site excluding SGPV16. The mean normalized heterozygosity +/– SD for all loci with the exclusion of SGPV16. The expected and observed heterozygosity for each locus was normalized by the maximum. Triangles are expected heterozygosity and circles are observed. Sample sites are arranged from left to right on the x-axis based on their geographic position, west to east. The Arctic subspecies is colored purple, the Baltic subspecies is maroon, and the Lake Saimaa subspecies is black. Note, Ulukhaktok/Holman is denoted as Holman. (TIFF) [file pone.0077125.s021.tif]

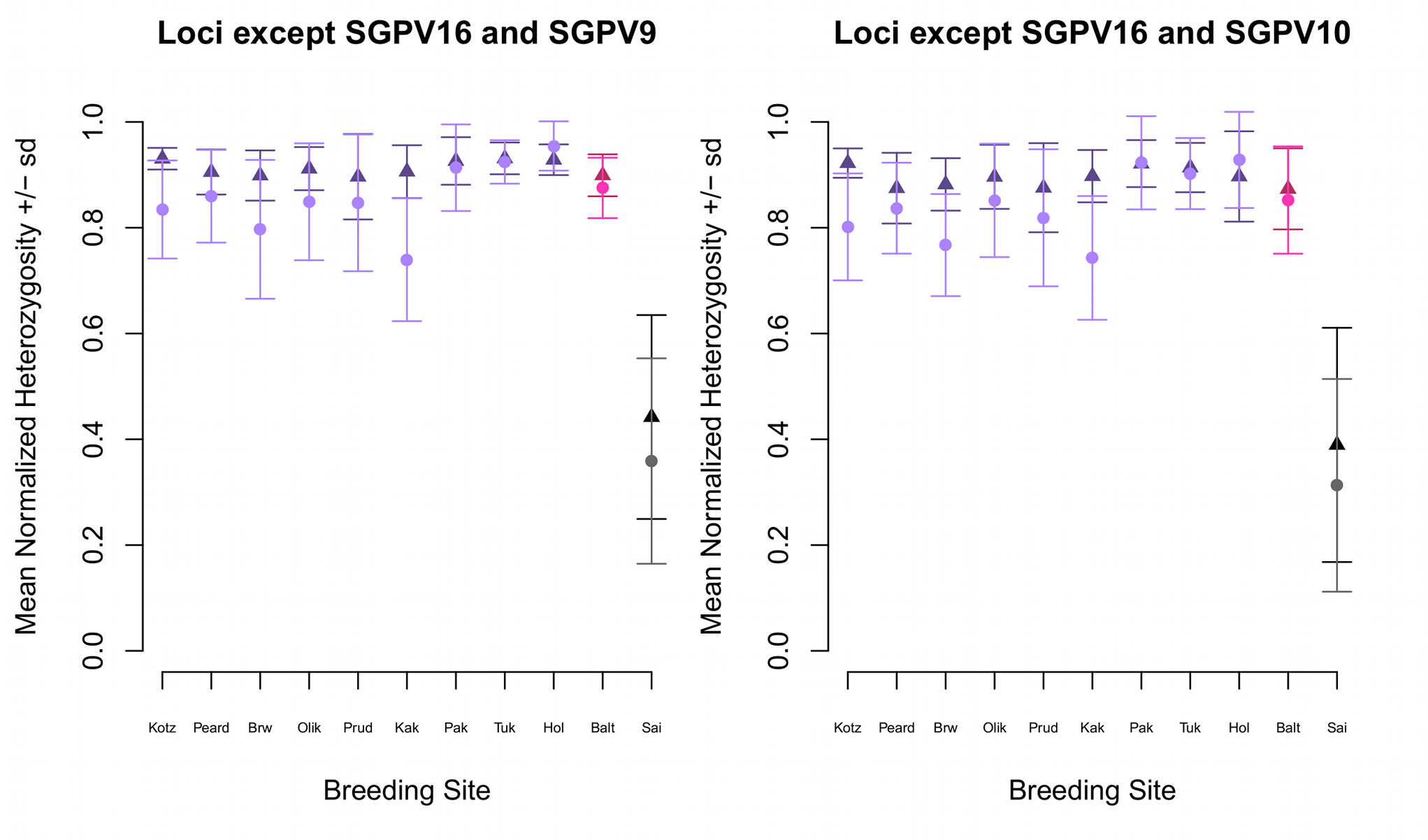

Supplement: Figure S22 — Normalized heterozygosity by sample site with SGPV16 and other loci excluded. The mean normalized heterozygosity +/– SD for all loci with the exclusion of SGPV16 and an additional locus. The expected and observed heterozygosity for each locus was normalized by the maximum. Triangles are expected heterozygosity and circles are observed. Sample sites are arranged from left to right on the x-axis based on their geographic position, west to east. The Arctic subspecies is colored purple, the Baltic subspecies is maroon, and the Lake Saimaa subspecies is black. Note, Ulukhaktok/Holman is denoted as Holman. (TIFF) [file pone.0077125.s022.tif]

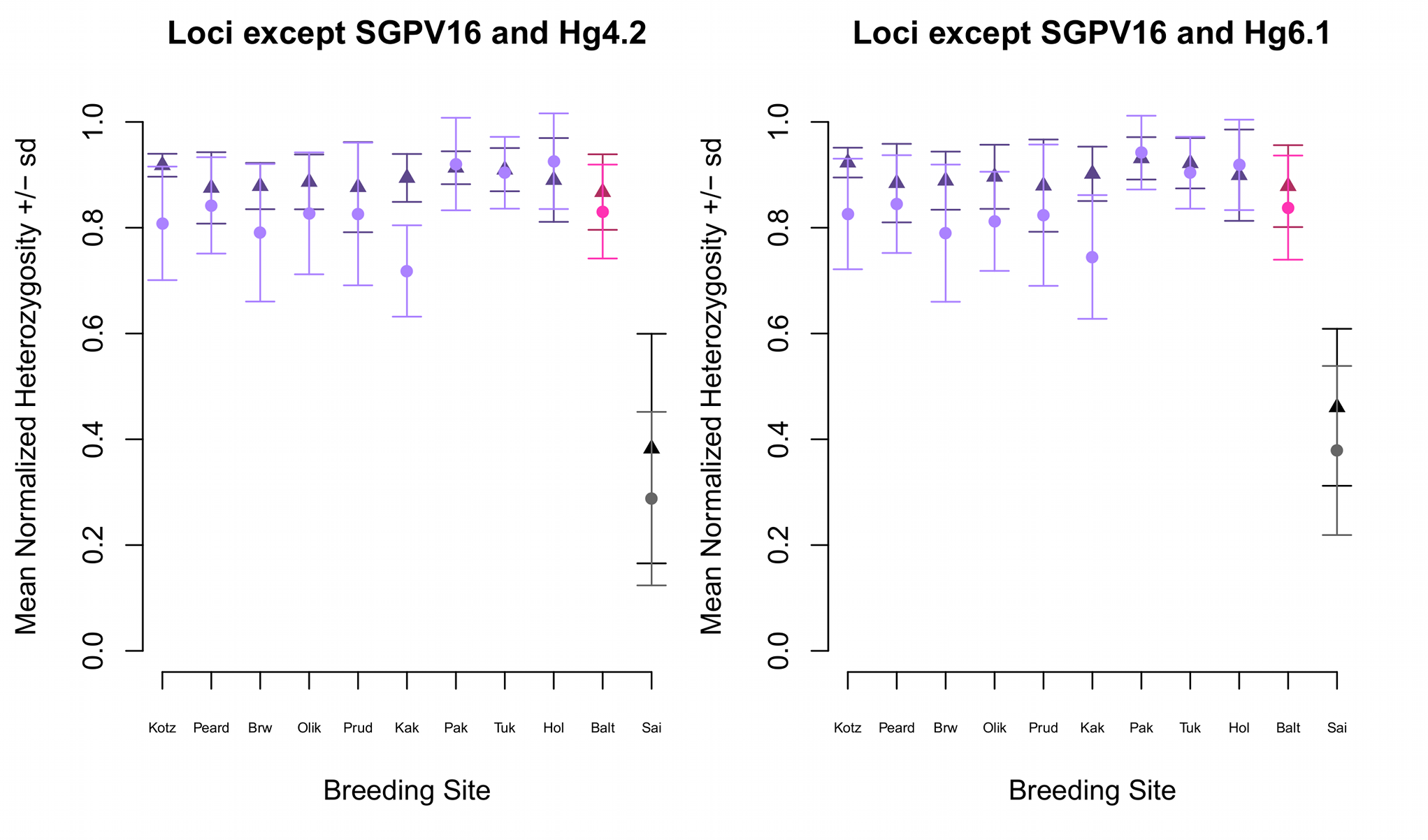

Supplement: Figure S23 — Normalized heterozygosity by sample site with SGPV16 and other loci excluded. The mean normalized heterozygosity +/– SD for all loci with the exclusion of SGPV16 and an additional locus. The expected and observed heterozygosity for each locus was normalized by the maximum. Triangles are expected heterozygosity and circles are observed. Sample sites are arranged from left to right on the x-axis based on their geographic position, west to east. The Arctic subspecies is colored purple, the Baltic subspecies is maroon, and the Lake Saimaa subspecies is black. Note, Ulukhaktok/Holman is denoted as Holman. (TIFF) [file pone.0077125.s023.tif]

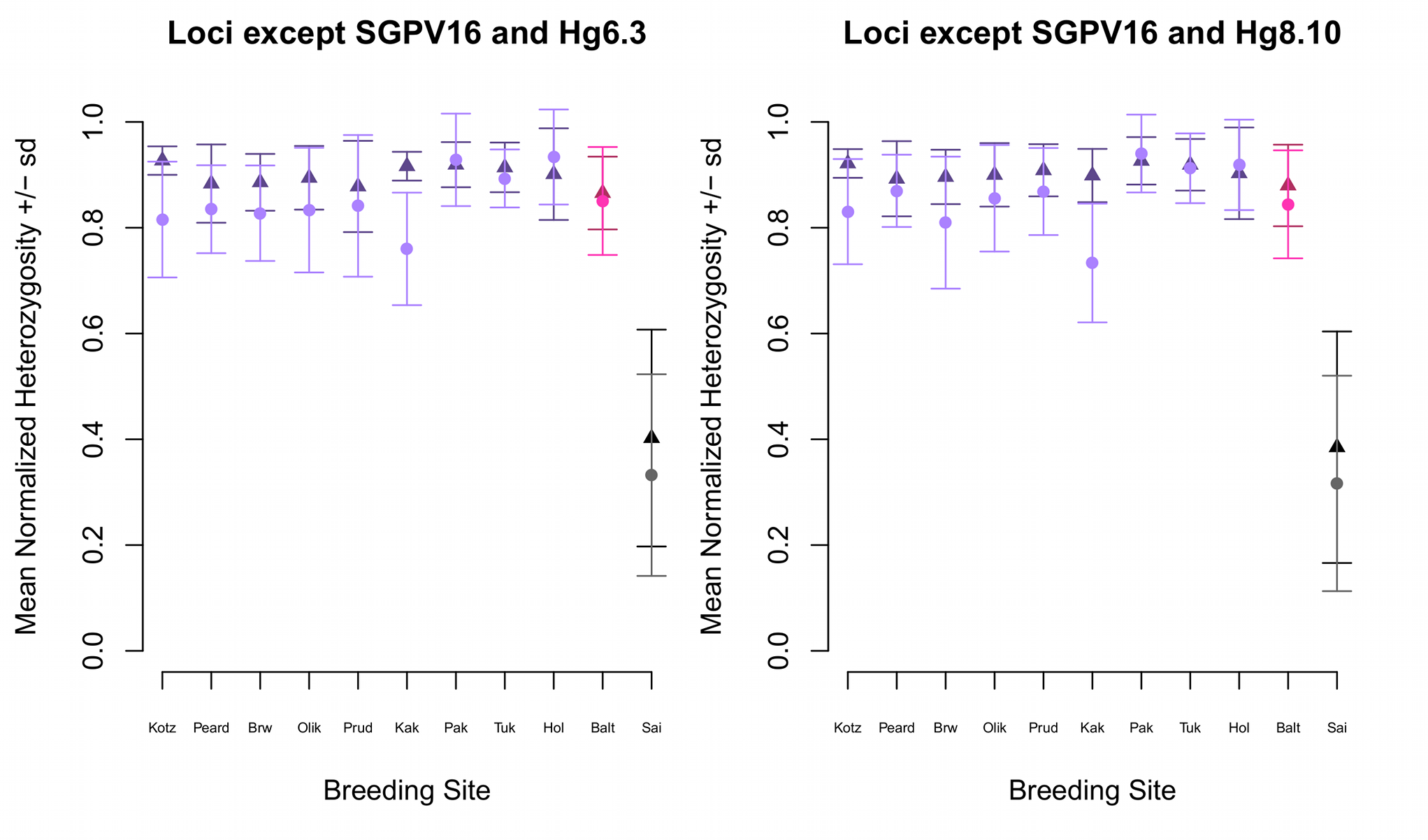

Supplement: Figure S24 — Normalized heterozygosity by sample site with SGPV16 and other loci excluded. The mean normalized heterozygosity +/– SD for all loci with the exclusion of SGPV16 and an additional locus. The expected and observed heterozygosity for each locus was normalized by the maximum. Triangles are expected heterozygosity and circles are observed. Sample sites are arranged from left to right on the x-axis based on their geographic position, west to east. The Arctic subspecies is colored purple, the Baltic subspecies is maroon, and the Lake Saimaa subspecies is black. Note, Ulukhaktok/Holman is denoted as Holman. (TIFF) [file pone.0077125.s024.tif]

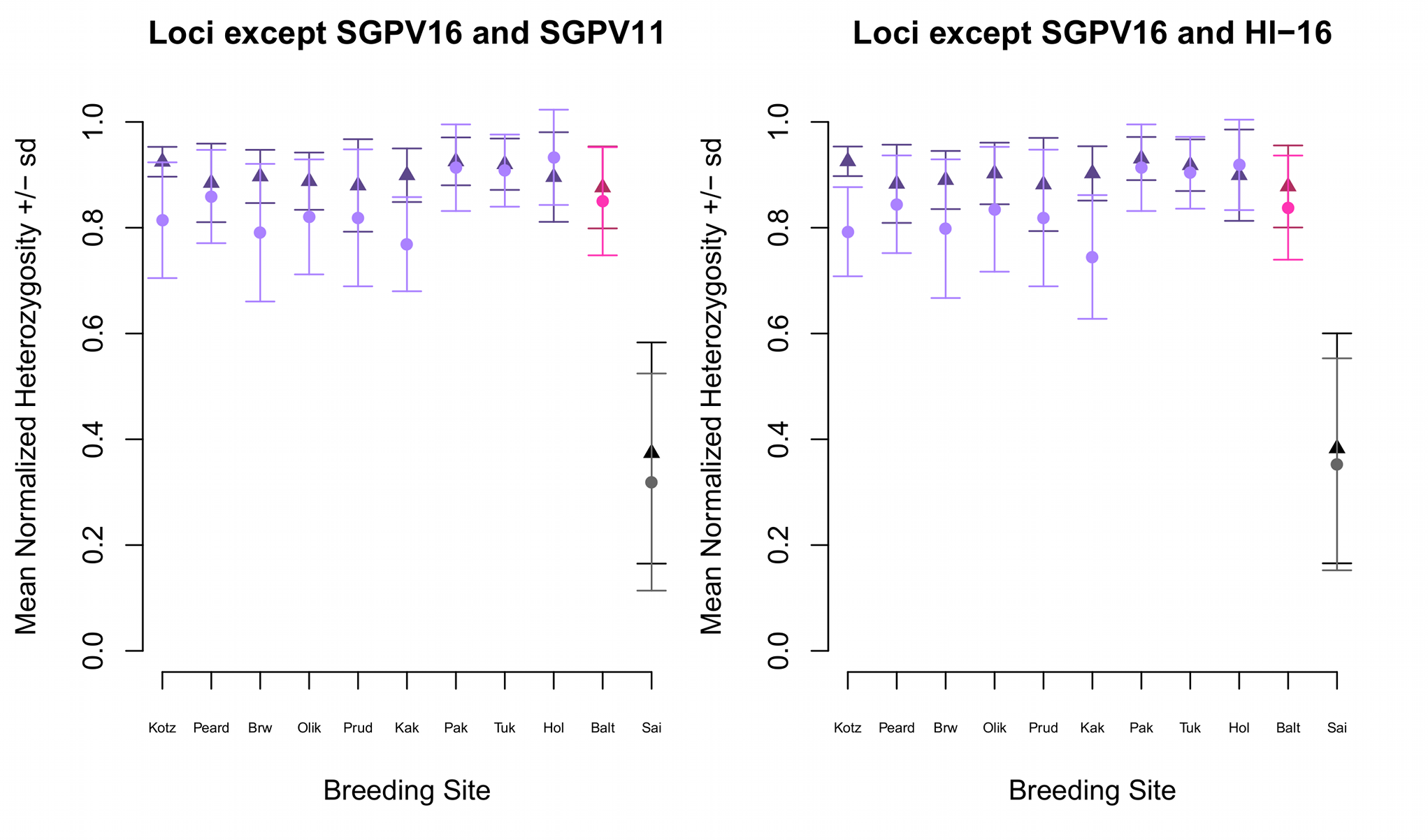

Supplement: Figure S25 — Normalized heterozygosity by sample site with SGPV16 and other loci excluded. The mean normalized heterozygosity +/– SD for all loci with the exclusion of SGPV16 and an additional locus. The expected and observed heterozygosity for each locus was normalized by the maximum. Triangles are expected heterozygosity and circles are observed. Sample sites are arranged from left to right on the x-axis based on their geographic position, west to east. The Arctic subspecies is colored purple, the Baltic subspecies is maroon, and the Lake Saimaa subspecies is black. Note, Ulukhaktok/Holman is denoted as Holman. (TIFF) [file pone.0077125.s025.tif]

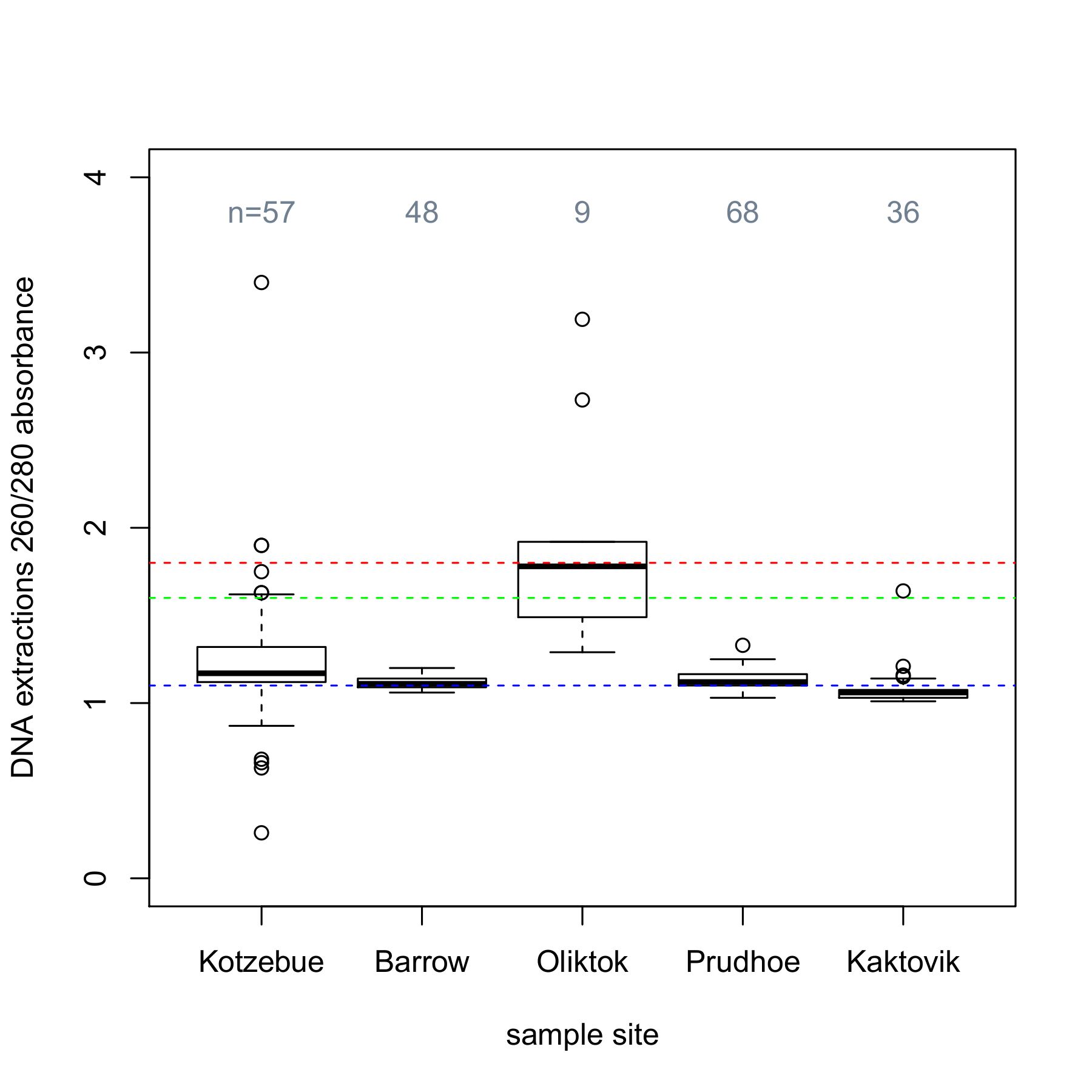

Supplement: Figure S26 — DNA extraction purity for shed-skin samples. Using a subset of the shed-skin samples collected in the Chukchi and Western Beaufort, DNA extraction quality was measured with an Eppendorf BioPhotometer. Boxplots show the distribution of the DNA purity by sample site. Pure DNA samples produce a 260/280 purity value of 1.8 (red line). A mean value of 1.6 (green) is typical for tissue samples and 1.1 (blue) for shed epidermis [25]. (TIFF) [file pone.0077125.s026.tif]

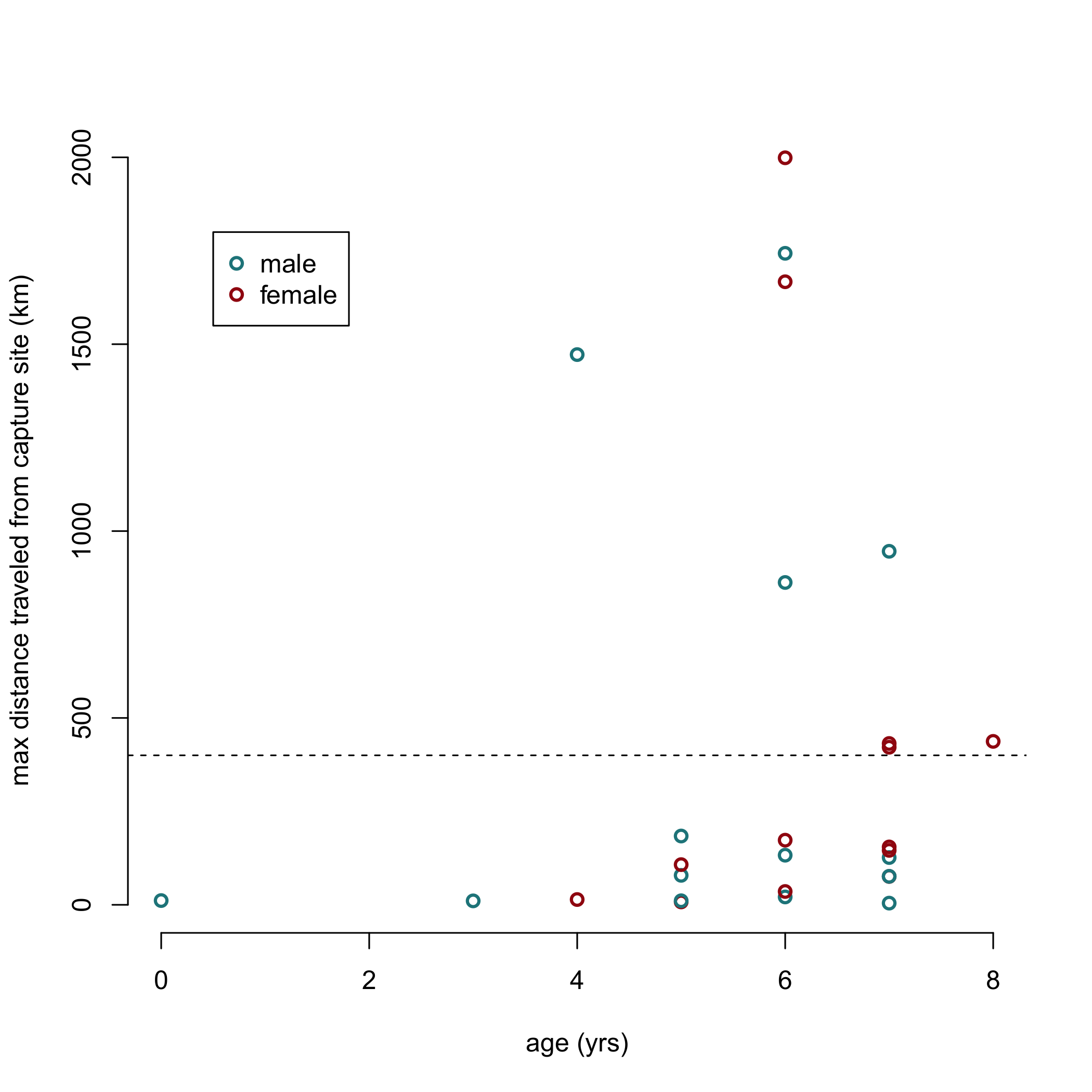

Supplement: Figure S27 — Travel vs. Age. Maximum distance travelled from capture site by age and sex. (TIFF) [file pone.0077125.s027.tif]
